# Ratiometric measurement of MAM Ca^2+^ dynamics using a modified CalfluxVTN

**DOI:** 10.1038/s41467-023-39343-2

**Published:** 2023-06-16

**Authors:** Eunbyul Cho, Youngsik Woo, Yeongjun Suh, Bo Kyoung Suh, Soo Jeong Kim, Truong Thi My Nhung, Jin Yeong Yoo, Tran Diem Nghi, Su Been Lee, Dong Jin Mun, Sang Ki Park

**Affiliations:** Department of Life Sciences, Pohang University of Science and Technology, Pohang, 37673 Republic of Korea

**Keywords:** Calcium, Organelles, Biosensors, Fluorescence imaging, Fluorescent proteins

## Abstract

Mitochondria-associated ER membrane (MAM) is a structure where these calcium-regulating organelles form close physical contact sites for efficient Ca^2+^ crosstalk. Despite the central importance of MAM Ca^2+^ dynamics in diverse biological processes, directly and specifically measuring Ca^2+^ concentrations inside MAM is technically challenging. Here, we develop MAM-Calflux, a MAM-specific BRET-based Ca^2+^ indicator. The successful application of the bimolecular fluorescence complementation (BiFC) concept highlights Ca^2+^-responsive BRET signals in MAM. The BiFC strategy imparts dual functionality as a Ca^2+^ indicator and quantitative structural marker specific for MAM. As a ratiometric Ca^2+^ indicator, MAM-Calflux estimates steady-state MAM Ca^2+^ levels. Finally, it enables the visualization of uneven intracellular distribution of MAM Ca^2+^ and the elucidation of abnormally accumulated MAM Ca^2+^ from the neurons of Parkinson’s disease mouse model in both steady-state and stimulated conditions. Therefore, we propose that MAM-Calflux can be a versatile tool for ratiometrically measuring dynamic inter-organellar Ca^2+^ communication.

## Abstract (web summary)

MAM Ca^2+^ dynamics play an important role in diverse biological processes, but directly and specifically measuring Ca^2+^ concentrations in this region is technically challenging. Here the authors report a MAM-specific BRET-based Ca^2+^ indicator called MAM-Calflux, which works as both a Ca^2+^ indicator and a structural marker due to its ratiometric nature.

## Introduction

Mitochondria and endoplasmic reticulum (ER) form close contact sites spanning ~10–30 nm and are called mitochondria-associated ER membranes (MAM) or mitochondria-ER contacts (MERC)^1,2^. MAM is a signaling hub that governs ER-mitochondria Ca^2+^ transfer^3–6^, lipid synthesis and exchange^7^, and mitochondrial structure dynamics^2^ to regulate autophagy^4,8^, ER stress response^9^, inflammasome activation^10^, and apoptosis^11^. Accumulating evidence implies that MAM is rearranged in response to diseases such as Parkinson’s disease (PD)^12,13^, Alzheimer’s disease (AD)^14,15^, amyotrophic lateral sclerosis (ALS)^16,17^, and type 2 diabetes mellitus (T2DM)^18^; yet the underlying mechanisms are largely unexplored.

MAM serves as a ground for dynamic Ca^2+^ transfer from the ER to mitochondria, thus playing an important role in diverse biological processes. Ca^2+^ indicators, which are located at the mitochondrial matrix or cytosolic part of the outer mitochondrial membrane (OMM), determined that, upon Ca^2+^ release from the ER, the mitochondria are then exposed to a higher concentration of Ca^2+^ compared with that in the cytosol; this microdomain with high-Ca^2+^ concentration is the MAM^3,14^. The MAM is concentrated with several proteins responsible for MAM Ca^2+^ transfer, including inositol triphosphate receptors (IP3Rs) and polycystin-2 (PKD2), which are involved in Ca^2+^ release from the ER, and voltage-dependent anion channels (VDACs), which are involved in Ca^2+^ influx into the mitochondria. In addition, the MAM structure is maintained by tethering proteins such as glucose-regulated protein 75 (GRP75) and DJ-1^1,13^, which also participate in regulating MAM Ca^2+^ transfer. Accumulated Ca^2+^ on the mitochondrial surface activates enzymes responsible for cellular bioenergetics and mitochondrial transport^9,19,20^. MAM Ca^2+^ transfer is also important for inhibiting autophagy^4,8^, stimulating Drp1-dependent mitochondrial division and pro-apoptotic signaling^2,21^, and buffering local cytosolic Ca^2+^ concentration^5^. Despite its importance, there is a limitation in the methodology used to directly and specifically measure MAM Ca^2+^, resulting in a poor understanding of MAM Ca^2+^ physiology.

Genetically encoded calcium indicators (GECIs) are powerful tools for understanding Ca^2+^-mediated cellular functions, particularly in cell biology and neuroscience. GCaMP, one of the most abundantly utilized GECI, is composed of a calmodulin-binding sequence of myosin light chain kinase (M13), circularly permutated green fluorescent protein (GFP), and calmodulin. The binding of Ca^2+^ to calmodulin and M13 induces conformational changes, which increase the GFP fluorescence intensity^22^. Over the past decades, significant progress has been made in the development of GECIs with longer wavelengths, higher signal-to-noise ratios, and faster kinetics^23,24^. In addition, ratiometric versions of GECIs were invented to overcome the limitations of GCaMPs whose basal signal intensities are multiplied in an expression-level-dependent manner. Ratiometric GECIs enable the quantitation of steady-state Ca^2+^ concentrations, such as in GCaMP-R based on the GCaMP/RFP ratio^25^, Twitch based on fluorescence resonance energy transfer (FRET)^26^, and Nanolantern or CalfluxVTN based on bioluminescence resonance energy transfer (BRET)^27^. Efforts have been made to understand intracellular Ca^2+^ distribution by developing organelle- or compartment-specific GECIs, such as for mitochondria^28^, ER^29^, and synapses^30^. However, limitations exist in directly and specifically investigating MAM Ca^2+^, and hence, indirect estimations are made by comparing Ca^2+^ dynamics separately measured from the ER and mitochondria^6,31^. Thus, the development of a MAM-specific Ca^2+^ sensor represents the effort to better understand the physiology of MAM Ca^2+^ dynamics.

In this study, we devise a modified version of CalfluxVTN which is specific for MAM, named MAM-Calflux. MAM-Calflux can directly probe MAM-specific Ca^2+^ concentration by applying bimolecular fluorescence complementation (BiFC). Venus fluorescence is only operative where the ER and mitochondria are closely tethered, allowing the MAM-specific observation of Ca^2+^-responsive BRET signals. The specificity of the Venus to the MAM also enables the use of this sensor as a quantitative structural marker, facilitating the dual functionality. MAM-Calflux demonstrates an uneven distribution of MAM Ca^2+^ dynamics. Furthermore, we apply this sensor to estimate MAM Ca^2+^ dynamics in the neurons of multiple neurodegenerative disease mouse models.

## Results

### MAM-Calflux, a BRET-based MAM-specific calcium indicator

**Fig. 1:**
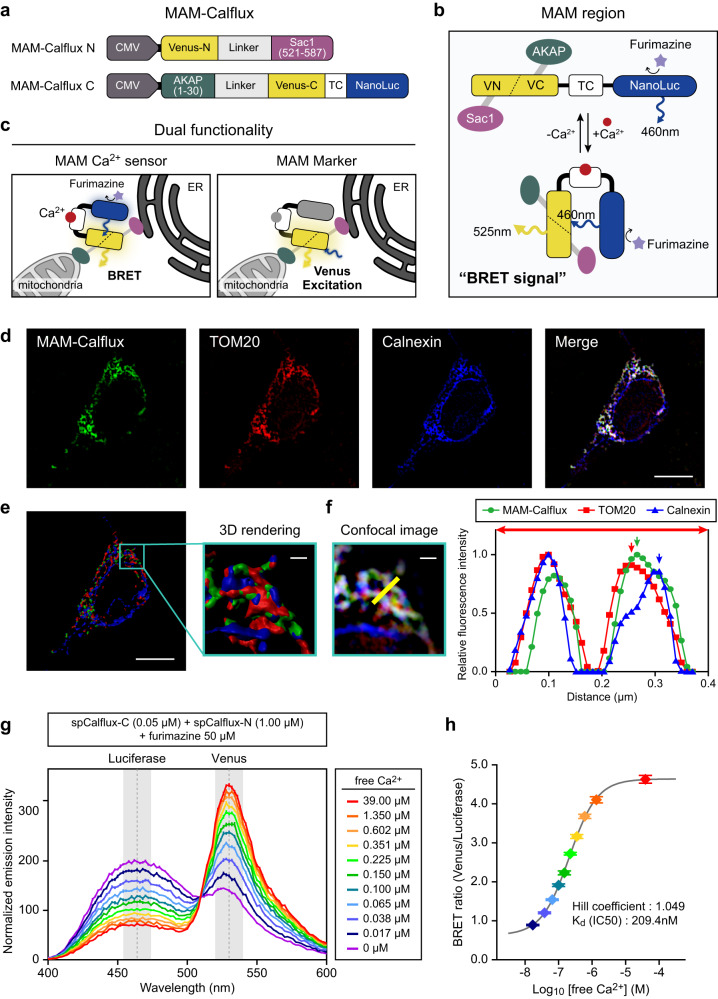
MAM-Calflux, a BRET-based MAM-specific calcium indicator. **a** Schematic design of MAM-Calflux constructs. Based on the CalfluxVTN construct, Venus was divided into two fragments, Venus-N (1–173 aa of Venus, VN) and Venus-C (156–228 aa of Venus, VC). Venus-N was conjugated with ER-targeting sequence (521–587 aa of Sac1) and Venus-C with troponin C (TC), and Nanoluciferase (NanoLuc) was conjugated with mitochondria-targeting sequence (1–30 aa of AKAP). **b** Schematic representation of the working principle of MAM-Calflux function depending on Ca^2+^ binding. At the MAM site, Venus-N and Venus-C associate with each other to form a functional fluorescence domain. NanoLuc emits a bioluminescence signal (460 nm) in the presence of the substrate (furimazine). When Ca^2+^ binds to TC, the conformational change results in drawing the fused Venus and NanoLuc into close proximity, followed by energy transfer and emission of the Venus signal (525 nm). **c** A dual functionality of MAM-Calflux. In addition to its application in Ca^2+^ sensing (left), the presence of functional Venus in the MAM region enables MAM-Calflux as a MAM structural marker through direct laser excitation of Venus (right). **d**–**f** Intracellular localization of MAM-Calflux. Transfected HeLa cells were immunostained with TOM20 and Calnexin as markers for mitochondria and ER, respectively (**d**). The 3D rendering image (**e**) and the line profile plot (**f**) represent MAM-Calflux localization between mitochondria and ER. Data representative of three experimental repeats. **g**–**h** Ca^2+^-dependent BRET from in vitro purified split-version of CalfluxVTN was measured using a microplate reader system. Luminescence wavelength scan (**g**) and the sigmoidal curve fitting of BRET ratios upon various free-Ca^2+^ concentrations (**h**). (*n* = 16, 16, 15, 16, 16, 12, 13, 16, 15, 16 microplate wells for 39.00, 1.350, 0.602, 0.351, 0.225, 0.150, 0.100, 0.065, 0.038, 0.017 μM free-Ca^2+^ groups, respectively, and *n* = 16 microplate wells for no Ca^2+^ group). The scale bars represent 10 μm for **d** and (**e**, left) and 1 μm for (**e**, right) and (**f**). All results for **g**, **h** are presented as mean ± SEM. Source data from **f**–**h** are provided as a Source Data file.

To establish a MAM-specific Ca^2+^ indicator, we applied the concept of bimolecular fluorescence complementation (BiFC)^32^ to a BRET-based Ca^2+^ indicator, CalfluxVTN^27^, with organelle targeting sequences. CalfluxVTN was separated into two fragments: 1–173 aa Venus fluorescence domain (VN173) and 156–228 aa Venus domain (VC155) conjugated with troponin C and Nanoluciferase (NanoLuc) domains. Linker sequences of optimized lengths were used to insert the ER-targeting sequence (Sac1 521–587 aa) and mitochondria-targeting sequence (AKAP 1–30 aa) into VN173 and VC155 fragments, respectively (Fig. 1a). According to the principle of BiFC assays, the Venus fragment is only fused at the sites where the mitochondrial and ER membranes are closely juxtaposed. This is to ensure that the BRET signal of MAM-Calflux is visible only at the MAM after binding of Ca^2+^ to troponin C, which triggers a conformational change due to the close apposition between NanoLuc and fused Venus. Thus, MAM-Calflux functions as a MAM-specific Ca^2+^ sensor (Fig. 1b). Additionally, after Venus is fused at the MAM, it can be visualized by the excitation-based fluorescence imaging instead of the bioluminescence- and Ca^2+^-dependent BRET. Therefore, MAM-Calflux can also be utilized as a MAM-specific structural marker using Venus fluorescence imaging without a NanoLuc substrate, thus displaying dual functionality (Fig. 1c).

First, we confirmed the organelle-specific localization of each of the two MAM-Calflux fragments. The expression of ER-targeted VN173 (MAM-Calflux-N) with the soluble form of VC155 resulted in a Venus fluorescent pattern that significantly overlapped with that of Sec61β-mCherry, an ER marker (Supplementary Fig. 1a, b). When mitochondria-targeted VC155 with troponin C and NanoLuc (MAM-Calflux-C) was transfected with the soluble form of VN173, Venus distribution significantly overlapped with that of TOM20, a mitochondrial marker (Supplementary Fig. 1c, d). Finally, co-expression of MAM-Calflux-N and MAM-Calflux-C resulted in the localization of Venus at the site where ER and mitochondrial markers overlapped, confirming the MAM-specific localization of MAM-Calflux (Fig. 1d–f). Since the self-assembly between fragments in the BiFC strategy was reported^33^, which can affect MAM formation, we optimized the experimental condition for the MAM-Calflux. MAM-Calflux expression did not affect MAM structures by 17 h after transfection (Supplementary Fig. 1e), but enhanced MAM structure by 48 h, providing optimal experimental time window (within 16–24 h after transfection) for measurement of MAM Ca^2+^ using MAM-Calflux.

To validate the Ca^2+^ response of MAM-Calflux, we measured the intensities of luminescence and BRET-based Venus fluorescence with or without Ca^2+^ using the spectrometry after treatment with furimazine, a substrate for NanoLuc, and ionomycin, a membrane-permeabilizing agent. In HeLa cells, either the co-expression of MAM-Calflux-N and MAM-Calflux-C or the single expression of MAM-Calflux-C resulted in a bioluminescent signal peak at 450–470 nm wavelength, confirming that NanoLuc functionality was maintained despite Venus cleavage (Supplementary Fig. 2a). MAM-Calflux had a significant emission peak at 520–530 nm wavelength, which was diminished by the Ca^2+^ chelator EGTA, indicating the existence of a Ca^2+^-responsive BRET signal (Supplementary Fig. 2a, b). MAM-Calflux-C, which contains intact NanoLuc but lacks the functional Venus, only had a bioluminescent signal without the BRET signal, implying that the BRET signal necessitates the fusion of Venus fragments. Rising BRET signals were observed as CaCl_2_ concentrations increased, confirming the capacity of MAM-Calflux to detect MAM Ca^2+^ (Supplementary Fig. 2c, d). The Ca^2+^ response kinetics of the sensor was also determined in vitro using purified proteins with various concentrations of free-Ca^2+^ via spectrometry (Fig. 1g, h).

Furthermore, we performed a microscopic image analysis of the BRET signal. Since the luminescence signal is much weaker than the fluorescence signal, we utilized an EMCCD camera with a beam splitter (dual-view) to acquire images of the bioluminescence (Em460) and BRET-based Venus (Em525) signals simultaneously in a single frame (Supplementary Fig. 3a–b). Since MAM-Calflux measures Ca^2+^ levels in MAM regions, we quantitated the BRET ratio after masking MAM regions based on Venus excitation signals (Supplementary Fig. 3c). Time-lapse imaging verified that MAM-Calflux stably emitted Em460 and Em525 signals after ~2 min of furimazine treatment, which was maintained for at least 10 min (Supplementary Fig. 3d). Notably, even though the Em525 signal was significantly detected, it was negligible compared with the excitation-based Venus fluorescent signal (Venus excitation) (Supplementary Fig. 4). This allowed us to simultaneously observe the MAM structure based on Venus excitation and the MAM Ca^2+^ concentration based on Em460/Em525 signals while the furimazine exists, emphasizing its dual functionality as a MAM marker and MAM Ca^2+^ indicator.

### MAM-Calflux visualizes the changes in MAM-calcium dynamics

**Fig. 2:**
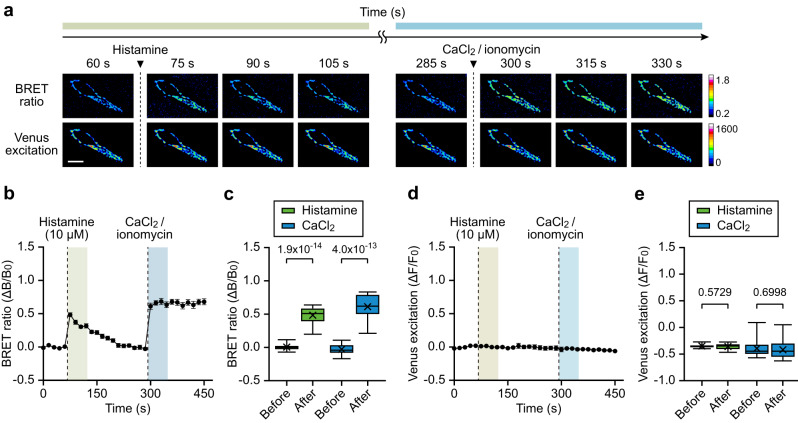
MAM-Calflux visualizes the changes in MAM-specific calcium dynamics. **a**–**e** Time-lapse imaging of MAM-Calflux with serial treatments of 10 μM histamine and 10 μM ionomycin with 1 mM CaCl_2_. Representative images (**a**) and time-dependent plots for BRET ratio (**b**) and Venus excitation (**d**). Bar graphs represent the BRET ratio (**c**) and Venus excitation intensities (**e**) before and after histamine and CaCl_2_/ionomycin treatments. (*n* = 15 cells for serial treatment group). The scale bar represents 20 μm. All results are presented as means ± SEM for **b**, **d** and box plots representing median and interquartile range with whiskers min/max value and the cross representing mean value for **c**, **e**. All *P* values were calculated using two-tailed Student’s *t* test comparing before and after each treatment for **c**, **e**. Source data from **b**–**e** are provided as a Source Data file.

Next, we attempted to validate the MAM-Calflux response to changes in MAM Ca^2+^. We treated the cells with histamine, which indirectly increases ER Ca^2+^ release through IP3Rs^3^, followed by CaCl_2_ and ionomycin, which directly induce excessive calcium influx. Time-lapse imaging detected an immediate increase in the BRET ratio (Fig. 2a–c). Meanwhile, Venus excitation was not affected by histamine and CaCl_2_/ionomycin, implying that the MAM structure was not significantly affected and the increased BRET ratio was specific for Ca^2+^ influx (Fig. 2d, e). The BRET ratio was restored within a few minutes after histamine treatment, while the increased BRET ratio after CaCl_2_/ionomycin treatment was maintained for >3 min (until the end of imaging). This indicates that the restored BRET ratio after histamine treatment presumably demonstrated active Ca^2+^ restoration in MAM.

### MAM-Calflux measures changes in the steady-state Ca^2+^ level at MAM

**Fig. 3:**
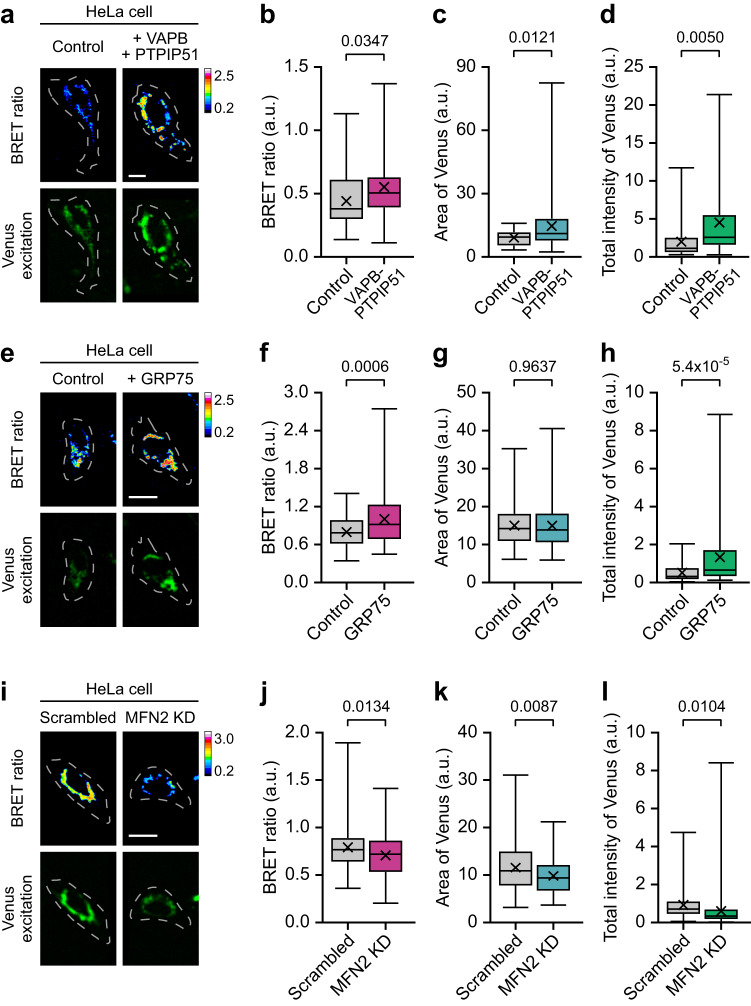
MAM-Calflux measures changes in the steady-state Ca^2+^ level at MAM. **a**–**d** Increased MAM Ca^2+^ levels and enhanced MAM structure formation measured by MAM-Calflux upon over-expression of VAPB-PTPIP51 tethering complex in HeLa cells. Representative images (**a**) and bar graphs representing BRET ratio (**b**), MAM area (**c**), and total Venus excitation intensities (**d**). Dashed lines in **a** represent the cell morphologies. (*n* = 45 for control HeLa cells, *n* = 33 for VAPB-PTPIP51 expressing HeLa cells). **e**–**h** Increased MAM Ca^2+^ levels and intact MAM structure measured by MAM-Calflux upon over-expression of GRP75 in HeLa cells. Representative images (**e**) and bar graphs representing BRET ratio (**f**), MAM area (**g**), and total Venus excitation intensities (**h**). (*n* = 68 for control HeLa cells, *n* = 82 for GRP75 expressing HeLa cells). **i**–**l** Decreased MAM Ca^2+^ levels and reduced MAM structure formation measured by MAM-Calflux upon knockdown of MFN2 in HeLa cells. Representative images (**i**) and bar graphs representing BRET ratio (**j**), MAM area (**k**), and total Venus excitation intensities (**l**). (*n* = 108 for control HeLa cells, *n* = 99 for MFN2 knockdown HeLa cells). Dashed lines in **a**, **e**, and **i** outline the cell morphologies. The scale bars represent 10 μm. All results are presented as box plots representing the median and interquartile range with whiskers min/max value and the cross representing the mean value. All P-values were calculated using two-tailed Student’s *t* test for **b**–**d**, **f**–**h**, and **j**–**l**. Source data from **b**–**d**, **f**–**h**, and **j**–**l** are provided as a Source Data file.

Compared with single fluorescent protein-based calcium indicators, including GCaMPs, FRET-based or BRET-based calcium indicators have the advantage that they are relatively independent from their expression levels because Ca^2+^ concentration is calculated as ratiometric signals^26^. This advantage allowed us to estimate steady-state MAM Ca^2+^ levels via MAM-Calflux imaging without any external Ca^2+^ stimulation. Thus, we introduced the conditions that were reported to regulate MAM contact size and examined the basal levels of the BRET ratio and Venus excitation of the MAM Ca^2+^ sensor and MAM marker, respectively. VAPB and PTPIP51 form a tethering complex that mediates the formation of the MAM structures^8,16^. The over-expression of VAPB and PTPIP51 increased both Venus excitation and BRET ratio in HeLa cells and primary cultured mouse neurons (Fig. 3a–d and Supplementary Fig. 5a–d), implying that both the MAM structure extent and MAM Ca^2+^ level, respectively, were increased by this tethering complex. In addition, VAPB-PTPIP51 expression increased histamine-induced Ca^2+^ influx in MAM and mitochondria without affecting cytosolic Ca^2+^ influx and ER Ca^2+^ release (Supplementary Fig. 5e–l and Supplementary Fig. 6). GRP75 is a chaperone tethering IP3R and VDAC1 mediating MAM Ca^2+^ crosstalk^1^. The BRET signals and total Venus intensities were upregulated by the over-expression of GRP75 (Fig. 3e–h), meaning that GRP75 increased MAM Ca^2+^ levels and MAM structure closeness but did not change the MAM structure area. This was recapitulated by staurosporine (STS) 2 h treatment (Supplementary Fig. 7), which induces early phase of apoptosis along with increased intracellular Ca^2+^ influx and MAM formation^34,35^. In contrast, the knockdown of a tethering protein MFN2 diminished basal MAM Ca^2+^ levels with low amounts of MAM structures (Fig. 3i–l). These results support the capacity of MAM-Calflux to determine changes in steady-state MAM Ca^2+^ level as well as in MAM structure.

### MAM-Calflux can determine the local distribution of MAM Ca^2+^ dynamics

Targeting GECIs to the mitochondrial surface helped visualize the uneven Ca^2+^ distribution on high-Ca^2+^ microdomains, which are largely relevant to the MAM^14,36^. In addition, local Ca^2+^ buffering at specific regions, especially the cell periphery and synapses, is regarded as an important role of mitochondria, ER, and MAM in maintaining their respective cellular functions^37–39^. However, whether MAM Ca^2+^ dynamics is locally determined independent of MAM structure has not yet been directly explored.

Based on the dual functionality of MAM-Calflux as a structural marker and a Ca^2+^ sensor, we hypothesized that MAM-Calflux can distinguish the intracellular localization of MAM structures and the distribution of Ca^2+^ concentrations among them. Line profile analysis in primary cultured neurons, which are highly polarized and necessitate the local distribution of organelles and Ca^2+^ concentration^40,41^, identified synchronized peak distributions of MAM Ca^2+^ intensity compared with that of MAM marker intensity (Supplementary Fig. 8a, b). Meanwhile, comparing MAM Ca^2+^ peak intensities between regions with corresponding MAM structural closeness, we found significant differences among them implying the local distribution of MAM Ca^2+^ dynamics among the MAM regions (Supplementary Fig. 8b). Furthermore, during time-lapse imaging of MAM-Calflux-expressing HeLa cells, we visualized the uneven distribution of Venus excitation and BRET ratio at the steady-state (pre-treatment) (Supplementary Fig. 8c). Notably, after histamine treatment, we observed that the BRET ratio was unevenly increased, and some MAM regions responded more strongly than others (Supplementary Fig. 8d), as previously suggested^37^. In contrast, the Venus excitation pattern was not largely affected by histamine, indicating that the uneven increase in the BRET signal was not induced by MAM structural changes (Supplementary Fig. 8e). Moreover, the direct Ca^2+^ influx due to CaCl_2_ increased Ca^2+^ signals in a general manner (Supplementary Fig. 8f, g), implying that the uneven increase of the BRET signal due to histamine could be attributed to the different IP3R-mediated Ca^2+^ responses between MAM regions.

**Fig. 4:**
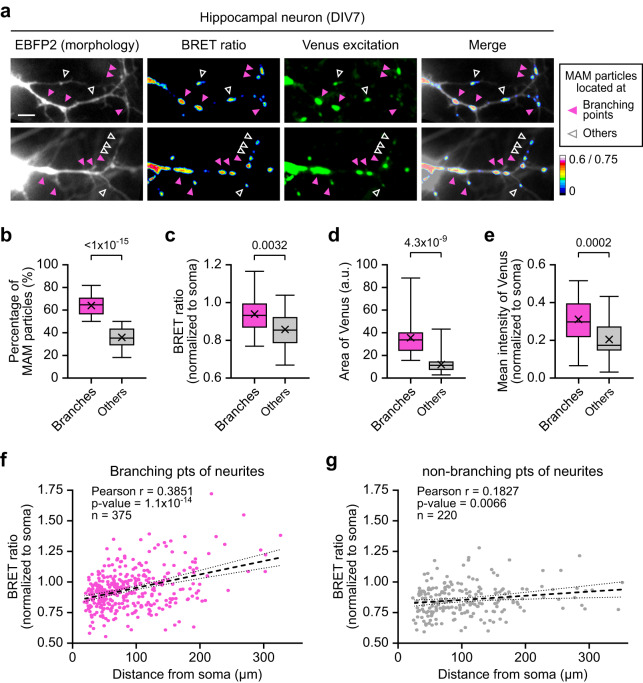
MAM-Calflux can determine the local distribution of MAM-specific calcium dynamics. **a**–**e** MAM-Calflux visualized the MAM puncta enriched at the branching points of developing neurites with higher Ca^2+^ levels. Representative images (**a**) for BRET signals upon the MAM puncta at the neurites of DIV7 hippocampal neurons. The magenta-filled arrowheads indicate MAM puncta at the branching points, and the empty white arrowheads indicate MAM puncta at the non-branching points. The fraction of MAM puncta at branching points in each neuron (**b**) was higher than the non-branching point. BRET ratio (**c**), MAM area (**d**), and mean Venus excitation intensities (**e**) of MAM puncta at the branching points were higher than others. (*n* = 27 neurons). **f**–**g** Correlation between BRET signals and distance from the center of soma among the MAM puncta at the branching points (**f**) and non-branching points (**g**). The scale bar represents 10 μm. All results are presented as box plots representing the median and interquartile range with whiskers min/max value and the cross representing the mean value. All *P* values were calculated using two-tailed Student’s *t* test for **b**–**e** and Pearson correlation coefficient analysis with two-tailed *P* values for **f**, **g**. For **f**, **g**, each dot represents each MAM puncta (*n* = 375 for branching points (**f**) and *n* = 220 for non-branching points (**g**) from 27 individual neurons). For **f**, **g**, black dashed lines represent simple linear regression with black dotted lines representing 95% confidence intervals. Source data from **b**–**g** are provided as a Source Data file.

Next, we analyzed MAM Ca^2+^ distribution in neuronal processes in DIV7 hippocampal neurons. We quantitated BRET signals in each MAM puncta and found a positive correlation between the BRET ratio and the distance from the soma (Supplementary Fig. 9). Moreover, approximately two-thirds of the MAM puncta in neurites were located at the branching points (Fig. 4a, b) and exhibited relatively higher Ca^2+^ levels and larger MAM areas (Fig. 4c–e). When we separately analyzed the correlation between the BRET ratio and the distance from the soma, MAM puncta at branching points had a stronger positive correlation than the ones at non-branching points (Fig. 4f, g). These results support that MAM accumulates at neurite branching points with pronounced Ca^2+^ dynamics that may be necessary properties such as local Ca^2+^ buffering^42,43^. These findings support the potential of MAM-Calflux in understanding the intracellular distribution of MAM structure and MAM Ca^2+^ dynamics in various cellular processes.

### MAM-Calflux can monitor altered MAM-calcium physiology in neurodegenerative disease model neurons

Recent studies suggest that neurodegenerative diseases, especially PD, AD, and ALS, are closely related to abnormal regulation of the MAM structure and thereby its functions. However, a deeper understanding of MAM Ca^2+^ physiology in these diseases is still required^44^. We introduced MAM-Calflux into the neurons of mouse models pertaining to these diseases to quantitate steady-state Ca^2+^ levels inside the MAM region, which was not previously achievable.

**Fig. 5:**
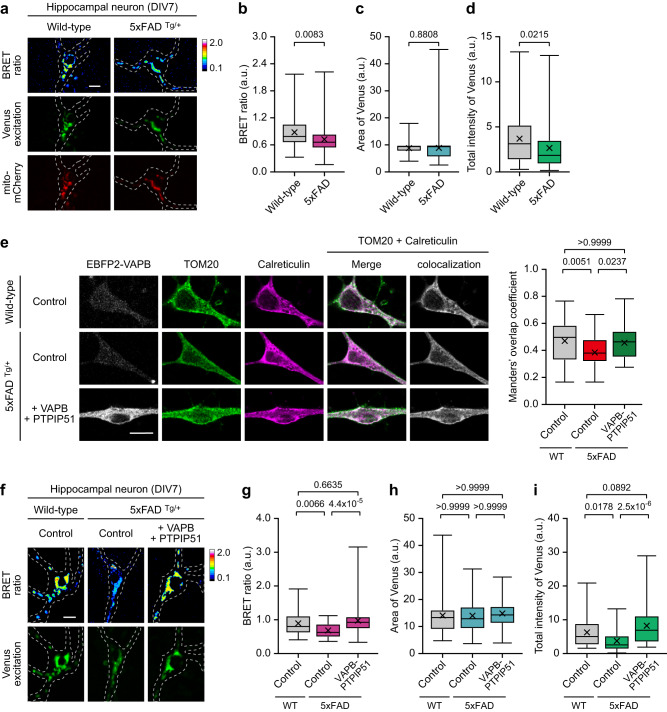
MAM-Calflux can monitor altered MAM structure and MAM Ca^2+^ levels in Alzheimer’s disease model neurons. **a**–**d** MAM-Calflux detected the decreased MAM Ca^2+^ levels and MAM integrity in the soma of primary cultured 5xFAD tg mouse neurons. Representative images (**a**) and bar graphs representing BRET ratio (**b**), MAM area (**c**), and total Venus excitation intensities (**d**). (*n* = 70 for wild-type neurons and *n* = 76 for 5xFAD tg neurons). **e** Representative images (left) and Manders’ overlap coefficients (right) of colocalization between mitochondria (TOM20) and ER (Calreticulin) among 5xFAD tg neurons with VAPB-PTPIP51 tethering complex over-expression. (*n* = 50 for wild-type neurons, *n* = 43 for 5xFAD tg neurons, and *n* = 48 for VAPB-PTPIP51 expressing 5xFAD tg neurons). **f**–**i** Representative images (**f**) and graphs representing BRET ratio (**g**), MAM area (**h**), and total Venus excitation intensities (**i**). (*n* = 48 for wild-type neurons, *n* = 54 for 5xFAD tg neurons, and *n* = 54 for VAPB-PTPIP51 expressing 5xFAD tg neurons). Dashed lines in **a** and **f** represent neuronal morphologies. The scale bars represent 10 μm. All results are presented as box plots representing the median and ianterquartile range with whiskers min/max value and the cross representing the mean value. All *P* values were calculated using two-tailed Student’s *t* test for **b**–**d** and one-way ANOVA with Bonferroni’s multiple comparison tests for **e** and **g**–**i**. Source data from **b**–**e** and **h**–**i** are provided as a Source Data file.

5xFAD is an AD mouse model that expresses disease-linked mutant forms of human APP and PSEN1^45^. At 7 days in vitro (DIV), the primary cultured 5xFAD hippocampal neurons had a decreased BRET ratio and Venus excitation intensities, indicating that both MAM Ca^2+^ levels and MAM extent were affected (Fig. 5a–d). Moreover, MAM Ca^2+^ levels were reverted in those neurons by expressing the VAPB-PTPIP51 complex, thereby reverting MAM structure, indicating that decreased steady-state MAM Ca^2+^ levels in part arising from loosened MAM (Fig. 5e–i). PINK1 (PTEN-induced kinase 1) is a serine/threonine kinase responsible for mitochondrial quality control, and its loss-of-function mutations cause autosomal recessive early-onset PD^46^; accumulating evidence indicates an association between PINK1 and MAM biology^47,48^. PINK1 KO neurons had decreased MAM Ca^2+^ concentrations, whereas MAM structure was not significantly altered (Supplementary Fig. 10a–d). *SOD1* (superoxide dismutase 1) is the second most frequent gene associated with familial ALS, and the accumulation of mutant SOD1 proteins in the MAM has been reported^17^. SOD1*G93A transgenic mouse neurons, which ectopically express human SOD1 with ALS-linked mutation, had a minimal effect on the Ca^2+^ dynamics and MAM structure (Supplementary Fig. 10e–h).

**Fig. 6:**
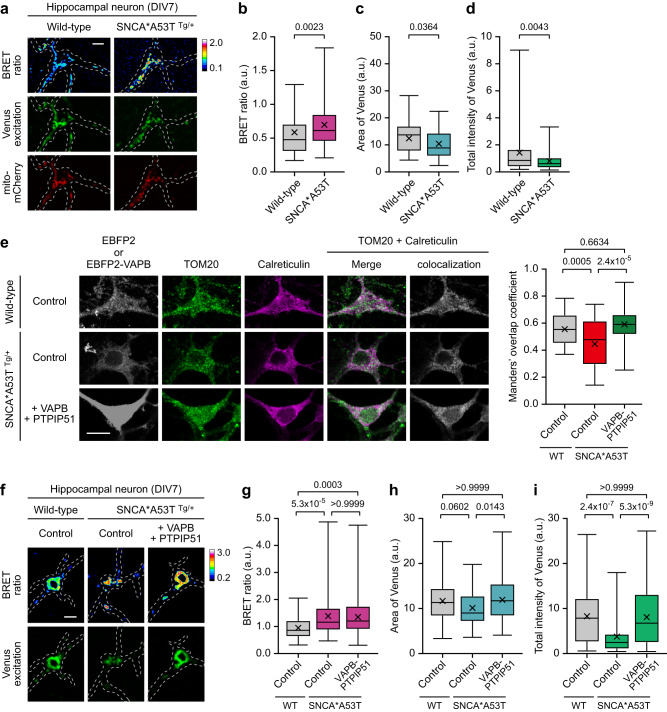
MAM-Calflux can determine the altered MAM-calcium physiology in the α-syn-mediated Parkinson’s disease model neurons. **a**–**d** MAM-Calflux detected the increased MAM steady-state Ca^2+^ levels with diminished structure integrity in primary cultured SNCA*A53T tg mouse neurons at DIV7. Representative images (**a**) and bar graphs representing BRET ratio (**b**), MAM area (**c**), and total Venus excitation intensities (**d**). (*n* = 71 for wild-type neurons and *n* = 66 for SNCA*A53T tg neurons). **e** Representative images (left) and Manders’ overlap coefficients (right) of colocalization between mitochondria (TOM20) and ER (Calreticulin) among SNCA*A53T tg neurons with VAPB-PTPIP51 tethering complex over-expression. (*n* = 55 for wild-type neurons, *n* = 43 for SNCA*A53T tg neurons, and *n* = 36 for VAPB-PTPIP51 expressing SNCA*A53T tg neurons). **f**–**i** Representative images (**f**) and graphs representing BRET ratio (**g**), MAM area (**h**), and total Venus excitation intensities (**i**). Dashed lines in **f** represent the neuronal morphologies. (*n* = 77 for wild-type neurons, *n* = 95 for SNCA*A53T tg neurons, and *n* = 92 for VAPB-PTPIP51 expressing SNCA*A53T tg neurons). Dashed lines in **a** and **f** represent the neuronal morphologies. The scale bars represent 10 μm. All results are presented as box plots representing the median and interquartile range with whiskers min/max value and the cross representing the mean value. All *P* values were calculated using two-tailed Student’s *t* test for **b**–**d** and one-way ANOVA with Bonferroni’s multiple comparison tests for **e** and **g**–**i**. Source data from **b**–**e** and **g**–**i** are provided as a Source Data file.

Alpha-synuclein (SNCA or α-syn) protein, which is located at the presynapse and regulates synaptic vesicles, is one of the most prominent causes of PD, as evidenced by its presence in Lewy bodies and missense mutations discovered in familial PD^49^. Ectopic expression of disease-linked SNCA mutants or excessive delivery of SNCA proteins has been reported to reduce ER-mitochondria contact and Ca^2+^ transfer^12,50^. When we examined MAM-Calflux in SNCA*A53T transgenic mouse neurons, which over-express the PD-linked A53T mutant form of human SNCA^51,52^, MAM steady-state Ca^2+^ levels were significantly enhanced (Fig. 6a, b). In contrast, the intensity of MAM structure was diminished (Fig. 6c, d), which is consistent with previous reports. BRET signals were not reverted by expressing the VAPB-PTPIP51 complex. However, it recovered Venus signals, indicating that increased steady-state MAM Ca^2+^ levels were largely independent of the loosened MAM structure (Fig. 6e–i). These results indicate that excessive α-syn proteins reduce the MAM structural extent, resulting in abnormal Ca^2+^ accumulation in MAM regions with possible link to PD pathogenesis.

## Discussion

Although Ca^2+^ transfer was assumed to be one of the major functions of MAM, specific and direct measurement of Ca^2+^ concentration in the MAM was not possible due to technical limitations. Instead, MAM Ca^2+^ has been evaluated by measuring cytosolic Ca^2+^ levels near the OMM^14,36^ or by comparing Ca^2+^ dynamics in the ER and mitochondria after stimulation of Ca^2+^ crosstalk^6,31^. Csordas et al. presented an advanced methodology utilizing rapamycin-inducible bridge-forming modules (RiBFM) to guide OMM-targeted Pericam toward the ER; thus, the signal was more specific to MAM, although this still could not exclude non-MAM Ca^2+^ signals and had a chance of artificial increase of MAM structures^53^. In this study, we modified the fluorescent domain of the ratiometric calcium indicator to be specifically located inside the MAM, which could directly and specifically measure steady-state MAM Ca^2+^ levels without external stimuli. Its ratiometric character enables the estimation of MAM Ca^2+^ physiology with cell-to-cell or region-to-region comparisons. For instance, when we compared steady-state BRET ratios of different cell types which were almost simultaneously evaluated, such as HeLa control groups and primary cultured hippocampal neuron control groups in Fig. 3a–d, Supplementary Fig. 5a–d, and Supplementary Fig. 7, the neuronal cells exhibited higher MAM Ca^2+^ signals than that of HeLa cells, although the MAM structure intensities (Venus excitation) were similar. This implies that MAM in neuronal cells contains more activated Ca^2+^ transfer machinery or a higher frequency of MAM Ca^2+^ crosstalk. Therefore, the availability of data on the MAM-Calflux BRET ratio values corresponding to Ca^2+^ concentrations is critical for the precise measurement of MAM Ca^2+^ concentrations. Further applications of MAM-Calflux, such as comparisons of MAM Ca^2+^ concentrations in various cellular compartments and in response to various extracellular conditions, would help comprehend MAM Ca^2+^ physiology.

The specific characteristics of the BRET-based calcium indicator coupled with the BiFC strategy have imparted the dual functionality in MAM-Calflux as a MAM Ca^2+^ indicator and MAM structural marker (Fig. 1c). While given the substrate, the bioluminescence signal and BRET-based Venus signal indicate MAM Ca^2+^ concentrations. Independent of the substrate, laser excitation-based fluorescent imaging of Venus specifically exhibits the MAM structure since the Venus BiFC fragments can be fused only at MAM. Moreover, we verified that the detected BRET-based Venus signal was negligible compared with that of the excitation-based Venus fluorescence signal; thus, the MAM Ca^2+^ level and MAM structure could be simultaneously analyzed even during time-lapse imaging. Taking advantage of this dual functionality, we confirmed the previously reported local distribution of MAM Ca^2+^ physiology^36,53^. Under basal conditions, the MAM Ca^2+^ level was distinct from the MAM marker intensity, especially in polarized cells such as neurons (Fig. 4 and Supplementary Fig. 8a, b). On the one hand, MAM puncta stalled at the neurite branching points of developing neurons with higher Ca^2+^ levels than those of nearby MAMs. Emerging evidence emphasizes the importance of local Ca^2+^ modulation for proper axon/dendrite arborization^54^ and the positioning of organelles and cytoskeletons at the branching points^42,43^. Therefore, our findings are likely to provide insights into the roles of the local MAM-mediated Ca^2+^ dynamics in neuronal process development. We also observed that some MAM regions exhibited particularly strong Ca^2+^ signals during histamine-stimulated ER Ca^2+^ release by IP3Rs (Supplementary Fig. 8c–e). This possibly supports previous findings that IP3R clusters exist for local retuning Ca^2+^ activity and that IP3R isoforms differentially regulate MAM and local Ca^2+^ transfer^55,56^. Therefore, we suggest that further investigation using MAM-Calflux can elucidate the local MAM Ca^2+^ physiology under various conditions independent of local MAM structure formation.

In addition, the dual functionality of MAM-Calflux could help distinguish whether the change in MAM Ca^2+^ concentration is caused by the MAM structural change or by other factors such as altered Ca^2+^ transporter activity. Since the MAM provides the physical site for Ca^2+^ crosstalk between the ER and mitochondria, it is plausible to expect that a tighter MAM structure resulting in greater MAM Ca^2+^ dynamics. When the MAM structure was enhanced by STS treatment or ectopic expression of tethering proteins, MAM-Calflux demonstrated an increase in the steady-state MAM Ca^2+^ level (Supplementary Fig. 7 and Fig. 3). In contrast, alterations in the Ca^2+^ machinery affect the MAM Ca^2+^ dynamics without affecting the MAM structure. For instance, the knockout of B-cell lymphoma-extra large (Bcl-xL), an activator of voltage-dependent anion-selective channel protein 1 (VDAC1), decreased IP3R-mediated ER-mitochondria Ca^2+^ transfer. In contrast, the MAM structure was not affected^57^. We introduced MAM-Calflux into SNCA*A53T mouse neurons, indicating that the steady-state MAM Ca^2+^ level was significantly higher than that of the wild-type, whereas the MAM structural intensity decreased (Fig. 6). These upregulated Ca^2+^ levels were not affected by VAPB-PTPIP51 expression, indicating that this is independent of the loosened MAM structure. Indeed, α-syn was reported to interact with and block VDAC1, reducing Ca^2+^ influx to mitochondria and leading to the accumulation of Ca^2+^ on the MAM side^58^. In addition, α-syn aggregation binds to sarco/endoplasmic reticulum Ca^2+^-ATPase (SERCA) and increases cytosolic Ca^2+^ level at a later stage^59^. Therefore, based on results from MAM-Calflux, we hypothesize that the pathogenic α-syn decreases Ca^2+^ uptake by mitochondria, resulting in the abnormal accumulation of Ca^2+^ in the MAM regardless of its loosened structure. Likewise, defining both MAM structure and MAM Ca^2+^ by the dual functionality of MAM-Calflux can provide more insights into understanding MAM Ca^2+^ physiology in various cellular or disease-relevant conditions.

The MAM-specific localization and sustained Ca^2+^ response of MAM-Calflux proves that this strategy can be employed in developing calcium indicators specific for other inter-organelle contacts by making appropriate substitutions for organelle-specific localization signals of MAM-Calflux and optimizing the linker lengths^60^. For instance, the plasma membrane (PM)-ER contact serves for the efficient transfer of extracellular Ca^2+^ to the ER lumen and activates ER Ca^2+^ release under specific conditions such as muscular contraction^61^. Thus, interrogating Ca^2+^ crosstalk between the PM and ER would be informative. Dolman et al. determined that the mitochondria may be responsible for the Ca^2+^ gradient in *trans*- and *cis*-Golgi by close contact with Golgi apparatus^62^. Since Ca^2+^ dynamics in the Golgi affect its structures and functions, especially secretory pathways and local Ca^2+^ buffering^63^, the interaction between mitochondria and the Golgi apparatus is another possible candidate to examine Ca^2+^ crosstalk. Calcium indicators specific to these inter-organelle structures may advance our understanding of organellar Ca^2+^ dynamics and organelle-to-organelle Ca^2+^ crosstalk within the cell.

Although the MAM-Calflux is a versatile tool for studying MAM-specific Ca^2+^ physiology, a number of further improvements can be made. First, the split fragments of Venus by BiFC have been reported to undergo spontaneous self-assembly, although this was reduced when each fragment was fused to other proteins^64,65^. Self-assembly of Venus in MAM-Calflux would ectopically enhance MAM structure formation, resulting in the over-estimation of MAM Ca^2+^ basal levels. Indeed, we observed that expression of MAM-Calflux for long periods can affect MAM structure formation, requiring careful design of experimental conditions, such as expression period and levels. This may be partially overcome by modifying the strategy for Venus fragmentation in MAM-Calflux, for instance, introducing mutations in Venus^66,67^, changing Venus split residues^68^, and utilizing dimerization-dependent fluorescent protein^69^. Employing low-expression promoters or inducible expression systems will also be beneficial. Also, a relatively low bioluminescence signal and BRET-based Venus signal can restrict the frame-to-frame interval of time-lapse imaging. In our imaging condition, we achieved the shortest interval of 5 sec to observe MAM Ca^2+^ dynamics against external stimuli. Still, this interval may be insufficient to precisely analyze Ca^2+^ dynamics in some conditions, indicating room for further improvement in conjunction with the conventional BRET system^27^.

## Methods

### Ethical Statement

All animal procedures were approved by the Institutional Animal Care and Use Committee (IACUC) of Pohang University of Science and Technology (POSTECH-2022-0085). All experiments were performed in accordance with the approved guidelines.

### Animals

Pregnant B6 mice used for primary hippocampal neuron culture were purchased from Hyochang Science (Daegu, South Korea). SNCA*A53T mice were a generous gift from Dr. Won-Jong Oh (Korea Brain Research Institute)^52^. PINK1-deficient mice were a generous gift from Dr. Xiaoxi Zhuang (Chicago University) and Dr. Sang Myun Park (Ajou University School of Medicine)^70^. 5xFAD mice were a generous gift from Dr. Kyong-Tai Kim (Pohang University of Science and Technology)^45^. SOD1*G93A mice^71^ were purchased from The Jackson Laboratory (Stock No. 002726) and maintained on a C57BL/6 background. The animals were group-housed under diurnal light conditions (12 hr light, 12 hr dark cycle) and had free access to food and water. (temperature 22 °C ± 2 °C, humidity 50% ± 5%).

### Antibodies and plasmids

Anti-TOM20 mouse monoclonal antibodies (Cat# ab56783, Abcam, and Cat# sc-17764, Santa Cruz Biotechnology), anti-Calnexin (Cat# ab22595, Abcam), and anti-Calreticulin (Cat# ab2907, Abcam) were used for immunocytochemistry experiments for 1:50–1:200. For immunostaining, Alexa Fluor 568 or 647 conjugated goat anti-mouse IgG (Cat# A-11004 and Cat# A-21236, Molecular Probes) and Alexa Fluor 647 conjugated goat anti-rabbit IgG (Cat# A-21244, Molecular Probes) were used as secondary antibodies for 1:200.

pT7-CalfluxVTN, which was a gift from Carl Johnson (Addgene plasmid # 83926)^27^, was served as the template to design MAM-Calflux. A 173 aa N-terminal fragment of Venus domain of CalfluxVTN, VN173, was conjugated with a 103 aa linker and the ER-targeting sequence (mSac1 521–587 aa) at its C-terminus. A fragment containing the C-terminal part of Venus, VC155, and following Troponin C and NanoLuc domains was conjugated with the mitochondria-targeting sequence (mAkap1 1–30 aa) and a 57 aa linker at its N-terminus. Sec61b-mCherry was a gift from Gia Voeltz (Addgene plasmid # 49155)^72^. pCMV R-CEPIA1er was a gift from Masamitsu Iino (Addgene plasmid # 58216)^29^. The core sequence of human MFN2 shRNA was GGAAGAGCACCGTGATCAATG^73^.

### Cell culture and transfection

HeLa (ATCC # CCL-2) cells were grown in Dulbecco’s Modified Eagle Medium (DMEM) (HyClone) supplemented with 10% (v/v) fetal bovine serum (FBS) (Gibco) and 1% penicillin/streptomycin (Gibco). The cell-line was authenticated using STR profiling method and tested negative for mycoplasma contamination. For imaging samples, ~1.5 × 10^5^ cells/mL of cells were seeded on the 18-mm or 25-mm cover glass in 12-well or six-well plates, respectively. The cells were transfected using transfection reagents, either polyethylenimine (PEI) or Lipofectamine 2000 (Thermo Fisher Scientific). For transfection in a 12-well plate, 300–400 ng of MAM-Calflux-P2A construct and each 400-600 ng of other plasmids, such as EBFP2-N1, EBFP2-hVAPB, FLAG-hPTPIP51, MYC-hGRP75, and pLL3.7-mRFP-MFN2 shRNA, were mixed in 100 μL Opti-MEM with 4 μL PEI or 1 μL Lipofectamine 2000.

Primary cultures of hippocampal neurons were established by isolating E16 B6 mouse embryo hippocampal tissues in Hanks’ balanced salt solution (HBSS) (Gibco) and dissociating the tissues in 0.25% trypsin (Sigma-Aldrich) and 0.1% DNase I (Sigma-Aldrich) for 10 min at 37°C. Cells were resuspended in Neurobasal medium (Gibco) supplemented with 10 mM HEPES (pH 7.4) and 10% (v/v) horse serum to a final cell concentration of 4.0 × 10^5^ cells/mL. The cells were plated on glass coverslips pre-coated with poly-D-lysine and laminin. After 2 h of plating, the cell medium was replaced with Neurobasal medium containing 2 mM glutamine, 2% (v/v) B27 supplement (Gibco), and 1% (v/v) penicillin/streptomycin. The neurons were transfected with Lipofectamine 2000, and the medium was replaced with the culture medium 2 h after transfection. For transfection in a 12-well plate, 500–600 ng of MAM-Calflux-P2A construct and each 800–1000 ng of other plasmids were mixed in 100 μL Opti-MEM with 2 μL Lipofectamine 2000.

### Immunocytochemistry

For immunocytochemistry, the cells were fixed with 4% paraformaldehyde in PBS or 4% paraformaldehyde and 4% sucrose in PBS for 20 min and washed with PBS for three times. Cells were permeabilized with 0.2% Triton-X-100 in PBS for 5 min and blocked with 5% goat serum or 5% BSA in PBS for >30 min. For protein staining, the cells were incubated with primary antibodies diluted in the blocking solution for 1 h at room temperature or overnight at 4°C, washed with PBS for three times, and treated with secondary antibodies diluted in the blocking solution for 1 h at room temperature. Cell images were acquired using FV3000 confocal laser scanning microscope (Olympus) and processed using cellSens 2.3 software (Olympus) and ImageJ 1.52p (Fiji) software (National Institute of Health)^74^. Images were deconvolved using the advanced constrained iterative (CI) algorithm-based deconvolution program of cellSens software. Colocalization between ER and mitochondrial markers was quantitated using cellSens software, Imaris 9.2 software (Bitplane), or Fiji software.

### BRET measurement using plate reader system

To obtain in vitro purified Calflux proteins, the His6-tagged split-version of CalfluxVTN (lacking targeting sequences and linkers in MAM-Calflux) was introduced to the BL21 strain and cultured at 37 °C in LB medium containing 100 mg/mL ampicillin, until an OD_600nm_ of 0.6 was achieved. Subsequently, a 16-hour induction was performed using 1 mM isopropyl β-D-1-thiogalactopyranoside (IPTG) at 16 °C, followed by cell harvesting via centrifugation and snap-freezing in liquid nitrogen. The resulting cell pellets were resuspended in lysis buffer (20 mM Tris pH 8.0, 100 mM NaCl, 2 mM EDTA, 1% Triton-X, protease inhibitor, 1 mg/ml lysozyme, pH 7.4), incubated for 30 min at 37 °C, and sonicated (5 min at 20% amplitude, 5 s on, 2 s off). Cellular debris was removed through centrifugation at 7000 × *g* for 15 min at 4 °C, and the resulting clear supernatant was combined with Ni-NTA agarose beads in phosphate-buffered saline (PBS), followed by a 4-hour incubation at 4 °C. The beads were subsequently washed with a washing buffer (20 mM Tris pH 8.0, 100 mM NaCl, 20 mM imidazole), and the His-tagged proteins were eluted using an elution buffer (20 mM Tris pH 8.0, 100 mM NaCl, 300 mM imidazole). Finally, the proteins were dialyzed in a dialysis buffer (20 mM Tris-HCl pH 8.0, 100 mM NaCl, 3% glycerol, and 1 mM DTT). In vitro purified Calflux-N term and Calflux-C term were mixed to calcium calibration buffer being 1 μM and 0.05 μM for final concentrations, respectively. Calcium calibration buffer was made by mix of Ca-EGTA (10 mM CaEGTA, 100 mM KCl, and 30 mM MOPS pH 7.2) and zero calcium buffer (10 mM K_2_EGTA, 100 mM KCl, and 30 mM MOPS pH 7.2).

For live cell samples, HeLa cells in six-well plate were transfected with MAM-Calflux-C or MAM-Calflux. After 6 h, the cells were detached using trypsin-EDTA, resuspended in complete DMEM, and transferred to a white-bottom 96-well plate. For emission wavelength scan, after 12-16 h, the medium was replaced with Ca^2+^-free HBSS (Cat# 14175-095, Gibco) with 2 μM ionomycin and either 5 mM EGTA or variable concentrations of CaCl_2_ from 0.001 to 100 mM for 10–15 min.

Luciferase was activated by treating the cells with 50 μM of furimazine (Cat# AOB36539, Aobious) substrate. After stabilization for 2 minutes, the emission intensities of wavelengths from 400 to 600 nm with 2 nm intervals were measured at 37 °C using a multi-functional microplate reader (Tecan Infinite M200 pro).

### BRET imaging using EMCCD equipped fluorescence microscope

HeLa or primary cultured neurons on the cover glass were transfected with MAM-Calflux and EBFP2-N1, EBFP2-hVAPB and FLAG-hPTPIP51, and Myc-hGRP75. For MFN2 knockdown, HeLa cells were transfected with pLL3.7-mRFP-MFN2 shRNA, moved to a cover glass after 24–30 h of transfection, and again transfected with MAM-Calflux after 24–30 h of seeding. After 18–24 h, the cover glass was transferred to a live imaging chamber, and the cell medium was replaced with Ca^2+^-free HBSS with 10 mM HEPES and 50 μM furimazine. To maintain a sufficient concentration of furimazine, at least 250 μL of buffer was used for the 18-mm coverslip. After 5 min of stabilization with furimazine, BRET imaging was performed with an inverted fluorescence microscope (IX71, Olympus) using ×20 (UApoN340 ×20/0.70 W infinite/0.17/FN22), ×40 (UPlanSApo ×40/0.95 infinite/0.11-0.23/FN26.5), and ×60 (UPlanSApo ×60/1.35 Oil infinite/0.17/FN26.5) objective lenses at 37 °C. To get a 525/460 nm ratio image, a dual-view beam splitter (Photometrics) was installed in front of the EMCCD camera (Cat# C9100-13, Hamamatsu). To obtain the BFP/RFP and Venus images separately, a triple dichroic mirror (59001bs, Chroma) with dual-view emission filters for 525 nm (AT525/30 m, Cat# 223164, Chroma) and for 460 nm and 600 nm (59003 m, Cat# 365631, Chroma) were used. Images were acquired using MetaMorph 7.7 software (Molecular Devices) with multi-dimensional acquisition for the Em525 and Em460 dual image, Venus excitation image, and BFP excitation image, sequentially. Each image was obtained at 690 kHz, 11×, gain 100, binning 1, 10 s for Em460/Em525 imaging and 690 kHz, 1×, 500 ms for Venus excitation imaging. These conditions prevented the crosstalk of signals from Em525 into Venus excitation images. The BFP excitation imaging was obtained at 2.75 MHz, 1×, 300 ms. The images were analyzed using Fiji with the use of “Template Matching” plug-in to align Em460 and Em525 images, “Subtract Background” function or “BRET Analyzer 1.0.7” plug-in to subtract background signals, and “BRET Analyzer 1.0.7” plug-in to produce BRET ratio images. To calculate mean BRET ratio after masking MAM regions, “Threshold” and “Analyze Particles” functions were used over Venus excitation images to determine MAM-specific region-of-interests (ROIs).

### Live Ca^2+^ imaging using MAM-Calflux or mitochondria-, cytosolic-, and ER-specific Ca^2+^ sensors

Live calcium imaging was performed as previously described, with some modifications^6,75^. To measure the Ca^2+^ responses of subcellular organelles, HeLa cells or primary cultured hippocampal neurons were transfected with MAM-Calflux and EBFP2-N1, cyto-GCaMP6s and R-CEPIA1er, or mito-GCaMP6s and R-CEPIA1er constructs on DIV6. The cells were loaded with Ca^2+^-free HBSS and sequentially exposed to 10 μM histamine and 10 μM Ca^2+^-free ionomycin (Cat# I9657, Sigma-Aldrich) with 1 mM CaCl_2_. For a single treatment of histamine, cells were loaded with HBSS containing 1 mM MgCl_2_ and 2 mM CaCl_2_. To maintain a sufficient concentration of furimazine during time-lapse imaging, at least 250 μL of buffer was used for the 18-mm coverslip, and images were acquired within 20–25 min.

BRET images were recorded in MetaMorph software with multi-dimensional acquisition and time-lapse functions for the Em525 and Em460 dual image, the Venus excitation image, and the BFP excitation image, sequentially. In total 25–35 frames were acquired, and imaging conditions and interval times were adjusted to finally obtain ~15 seconds between frames. After image processing, the amplitude (Δ*B*/*B*_0_) of each neuron was calculated as (*B*−*B*_0_)/*B*_0_, where *B*_0_ is the baseline BRET ratio signal averaged over five frames before stimulation and B is the peak intensity of the BRET ratio in the response.

The fluorescence intensities were recorded at 37 °C with supplying 5% CO_2_ gas in FV31S-DT software at intervals of 2 seconds for 200 seconds in total using FV3000 confocal laser scanning microscope (Olympus) with UPLSAPO ×20/0.75 NA objective. Background fluorescence was subtracted and the amplitude (Δ*F*/*F*_0_) of each neuron was calculated as (*F*−*F*_0_)/*F*_0_, where *F*_0_ is the baseline mito-GCaMP6s, cyto-GCaMP6s, or R-CEPIA1er fluorescence signal averaged over 20 seconds before the stimulation and *F* is the peak intensity of fluorescence in the response.

### Statistics and reproducibility

All graphs are presented as the mean ± SEM or box plots representing the median and interquartile range with whiskers min/max value and the cross representing the mean value. The statistical significance of the data was analyzed using two-tailed Student’s *t* test for comparisons between two groups and one-way ANOVA followed by Bonferroni’s post-hoc test for comparisons among multiple groups. All statistical analysis, correlation analysis, and fitting with a sigmoidal curve model or a simple linear regression model were calculated using Prism 9.4 (GraphPad).

Sample sizes for all statistical evaluations are indicated in Figure legends. No statistical method was used to predetermine sample size. Number of cells per group was chosen based on sample size in the calcium measurement experiment with microscopic images which meets or exceed previous studies^6,60^. No data were excluded from the analyses. All sample images were acquired in random order within the sets and all cells were chosen at the random site. Experimenters were blinded to each group allocation during the image analysis by removing any sample information in file names.

### Reporting summary

Further information on research design is available in the Nature Portfolio Reporting Summary linked to this article.

## Supplementary information

Supplementary Information

Peer Review File

Reporting summary

## Data Availability

All data are available in the main text or the supplementary materials. Source data for all figures are provided with the paper. Materials are available from the corresponding author. Source data are provided with this paper.

## Source data

Source data
