# Supplementary material for: Ratiometric measurement of MAM Ca2+ dynamics using a modified CalfluxVTN
Source: Nat Commun. 2023 Jun 16;14:3586. doi: 10.1038/s41467-023-39343-2 (PMC10276021; doi:10.1038/s41467-023-39343-2)
Supplement: Supplementary file 1 — Supplementary Information [file 41467_2023_39343_MOESM1_ESM.pdf]

Supplementary Information

Ratiometric measurement of MAM Ca^{2+} dynamics using a modified CalfluxVTN

Eunbyul Cho, Youngsik Woo*, Yeongjun Suh, Bo Kyoung Suh, Soo Jeong Kim, Truong Thi My Nhung, Jin Yeong Yoo, Tran Diem Nghi, Su Been Lee, Dong Jin Mun, and Sang Ki Park*

* Correspondence: youngsik.woo@postech.ac.kr (Y.W.) and skpark@postech.ac.kr (S.K.P)

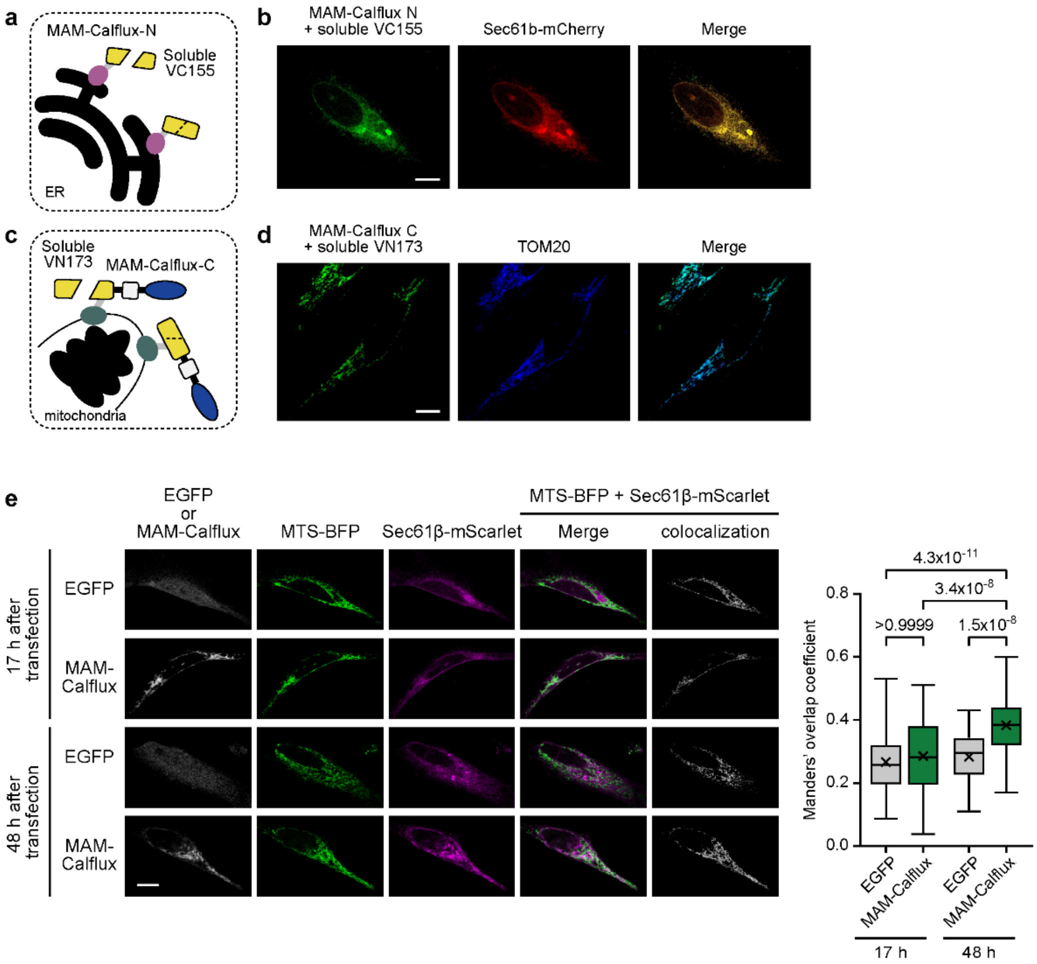

Supplementary Fig. 1. Validation of organellar localization of each MAM-Calflux N- and C-fragments.

(a-b) Venus fluorescent signals of MAM-Calflux N-fragment with soluble VC155 were co-localized with ER marker (Sec61β-mCherry), indicating the ER-specific localization of MAM-Calflux-N. Schematic diagram (a) and representative images (b). Data representative of three experimental repeats.

(c-d) Venus fluorescent signals of MAM-Calflux C-fragment with soluble VN173 were co-localized with an immunostained mitochondria marker (TOM20) indicating the mitochondria-specific localization of MAM-Calflux-C. Schematic diagram (c) and representative images (d). Data representative of three experimental repeats.

(e) Representative images (left) and Manders' overlap coefficients (right) of colocalization between

mitochondria (MTS-BFP) and ER (Sec61β-mScarlet) among over-expression of MAM-Calflux. (n = 78 HeLa cells for each group)

The scale bars represent 10 μm. All results are presented as box plots representing the median and interquartile range with whiskers min/max value and the cross representing the mean value. All P-values were calculated using one-way ANOVA with Bonferroni's multiple comparison tests for (e). Source data from (e) are provided as a Source Data file.

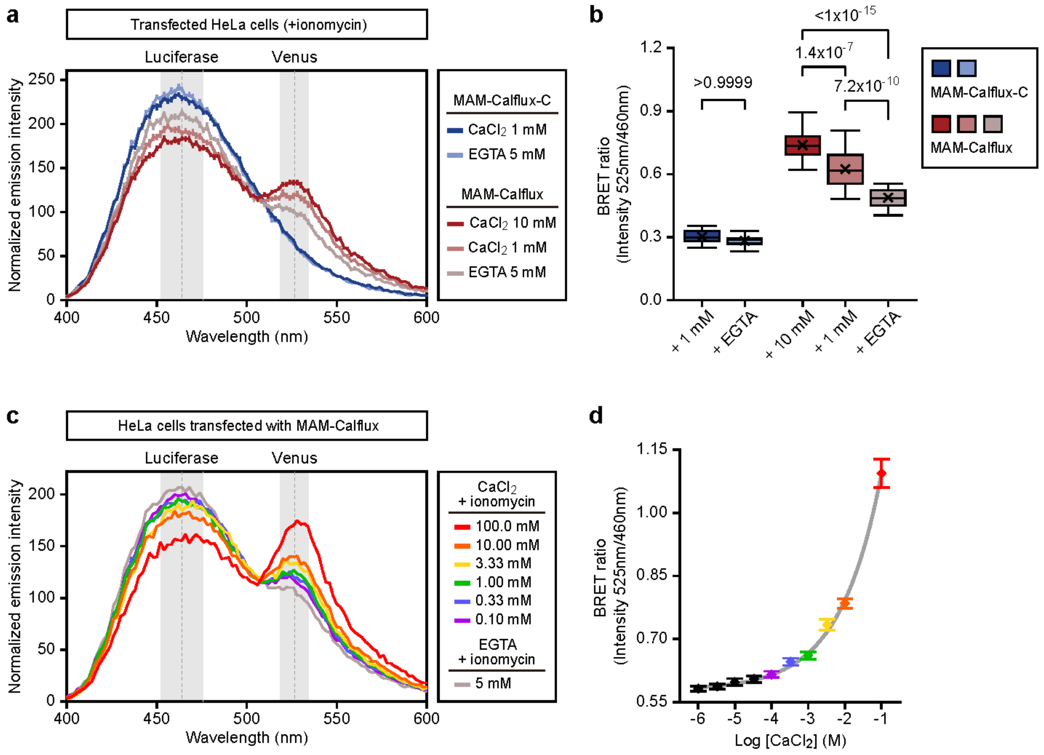

Supplementary Fig. 2. Validation of calcium-responsive BRET signals using a microplate reader system.

(a-b) Ca^{2+}-dependent BRET emission by MAM-Calflux from ionomycin-permeabilized HeLa cells measured using a microplate reader system. Luminescence wavelength scan (a) and calculation of BRET ratio (b) confirmed that the BRET-based Venus signal emission depends on the Ca^{2+} concentrations. The BRET ratio was calculated by dividing the mean emission intensities between 520–530 nm by those between 450–470nm. (n = 18 microplate wells for MAM-Calflux-C + $CaCl_2$ 1 mM group, n = 20 microplate wells for MAM-Calflux-C + EGTA 5 mM group, n = 20 microplate wells for MAM-Calflux-N+C + $CaCl_2$ 10 mM group, n = 18 microplate wells for MAM-Calflux-N+C + $CaCl_2$ 1 mM group, and n = 20 microplate wells for MAM-Calflux-N+C + EGTA 5 mM group)

(c-d) Luminescence wavelength scan of MAM-Calflux upon various concentrations of $CaCl_2$ or EGTA treatment with ionomycin (c) and the curve fitting of BRET ratios against Ca^{2+} concentrations (d). The solid gray line represents a sigmoidal curve fitting by Prism software. (n = 24 microplate wells for $CaCl_2$

100.0 mM group, n = 44 microplate wells for $CaCl_2$ 10.00 mM group, n = 24 microplate wells for $CaCl_2$ 3.33 mM group, n = 50 microplate wells for $CaCl_2$ 1.00 mM group, n = 44 microplate wells for $CaCl_2$ 0.33 mM group, n = 50 microplate wells for $CaCl_2$ 0.10 mM group, n = 50 microplate wells for $CaCl_2$ 0.03 mM group, and n = 50 microplate wells for $CaCl_2$ 0.01 mM group)

All results are presented as mean ± SEM for (a) and (d), and box plots representing the median and interquartile range with whiskers min/max value and the cross representing the mean value for (b). All P-values were calculated using one-way ANOVA with Bonferroni's multiple comparison test for (b). Source data are provided as a Source Data file.

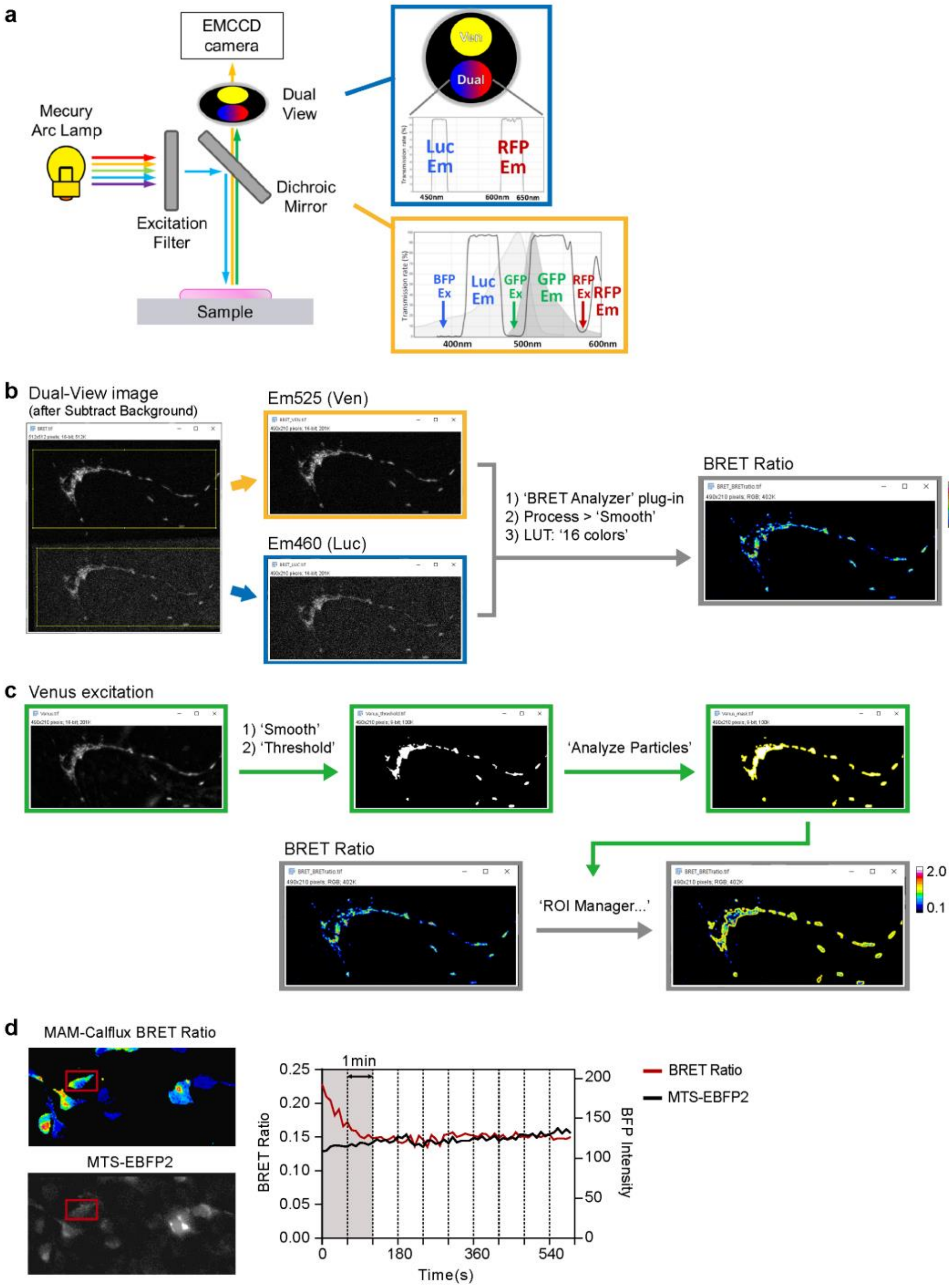

Supplementary Fig. 3. Overview and validation of microscopy set-up with the dual view and EMCCD camera for BRET imaging.

(a) Overview of microscopy set-up. EMCCD camera, beam splitter, dual-view, and triple dichroic mirror were used to obtain luciferase (donor) and BRET-based Venus (acceptor) images simultaneously

but separately. The dual view contains two filters that penetrate Luciferase/RFP emission signals (Dual) and Venus emission signal (Ven).

(b) Overview of image processing steps to prepare BRET ratio images.

(c) Overview of image analysis steps to measure BRET ratio upon Venus fluorescence signals.

(d) MAM-Calflux's furimazine stability test. Red rectangles indicate the region-of-interest for curve fitting (left). Intensity change in MAM-Calflux BRET ratio and MTS-EBFP2 with time after 50 μM furimazine treatment (right).

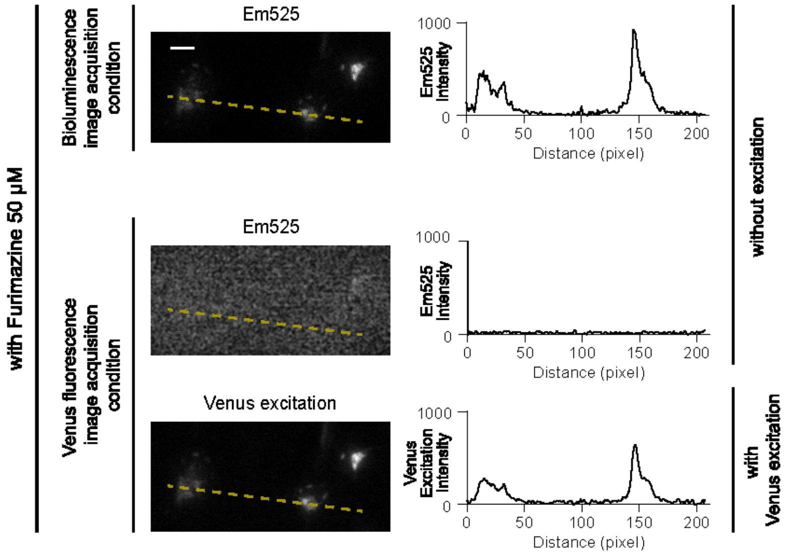

Supplementary Fig. 4. Comparison of BRET and fluorescence-based Venus signal intensities from MAM-Calflux imaging.

Test for crosstalk from the Em525 signal in the Venus excitation signal. Representative images and line scan plots of MAM-Calflux-expressing cells against Em525 (top), signal under Venus excitation setting without an excitation laser (middle), and Venus excitation (bottom). During the furimazine substrate-treated condition, although the BRET-based Venus signal (Em525, top) exists, it was almost negligible from the excitation-laser-based Venus signal (Venus excitation, bottom) when the BRET signal was acquired using Venus fluorescence imaging condition (middle). Data representative of four experimental repeats. The scale bars represent 20 μm.

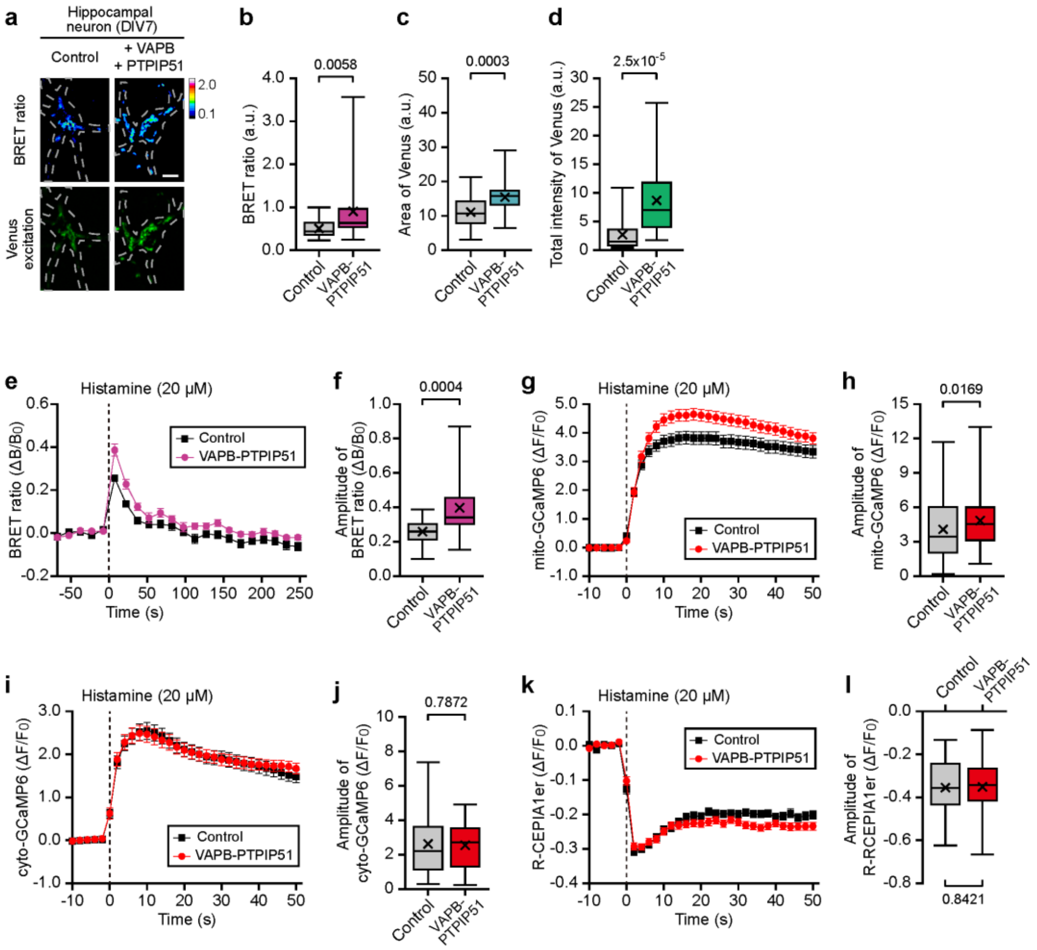

Supplementary Fig. 5. Validation of VAPB-PTPIP51 over-expression effects on MAM, mitochondrial, cytosolic, and ER Ca^{2+} dynamics.

(a-d) Increased MAM Ca^{2+} levels and enhanced MAM structure formation measured by MAM-Calflux upon the over-expression of the VAPB-PTPIP51 tethering complex in mouse hippocampal neurons. Representative images (a) and bar graphs representing BRET ratio (b), MAM area (c), and total Venus excitation intensities (d). Dashed lines in (a) represent the cell morphologies. (n = 29 for control neurons, and n = 31 for VAPB-PTPIP51 expressing neurons)

(e-f) Increased MAM Ca^{2+} influx was measured by MAM-Calflux in response to 20 µM histamine in VAPB-PTPIP51 expressing HeLa cells. Ca^{2+} response graph (e) and statistically analyzed peak amplitudes (f). (n = 26 for control and n = 37 for VAPB-PTPIP51 expressing HeLa cells)

(g-h) Increased mitochondrial Ca^{2+} influx was measured by mito-GCaMP6 in response to 20 μM histamine in VAPB-PTPIP51 expressing HeLa cells. Ca^{2+} response curve (g) and statistically analyzed peak amplitudes (h). (n = 128 for control and n = 118 for VAPB-PTPIP51 expressing HeLa cells)

(i-j) Comparable cytosolic Ca^{2+} influx was measured by cyto-GCaMP6 in response to 20 μM histamine in VAPB-PTPIP51 expressing HeLa cells. Ca^{2+} response graph (i) and statistically analyzed peak amplitudes (j). (n = 87 for control and n =50 for VAPB-PTPIP51 expressing HeLa cells)

(k-l) Comparable ER Ca^{2+} efflux was measured by R-CEPIA1er in response to 20 μM histamine in VAPB-PTPIP51 expressing HeLa cells. Ca^{2+} response graph (k) and statistically analyzed peak amplitudes (l). (n = 143 for control and n = 157 for VAPB-PTPIP51 expressing HeLa cells)

The scale bars represent 10 μm. All results are presented as means ± SEM for (e), (g), (i), and (k) and box plots representing the median and interquartile range with whiskers min/max value and the cross representing the mean value for (b-d), (f), (h), (j), and (l). All P-values were calculated using two-tailed Student's t-test for (b-d), (f), (h), (j), and (l). Source data from (b)-(l) are provided as a Source Data file.

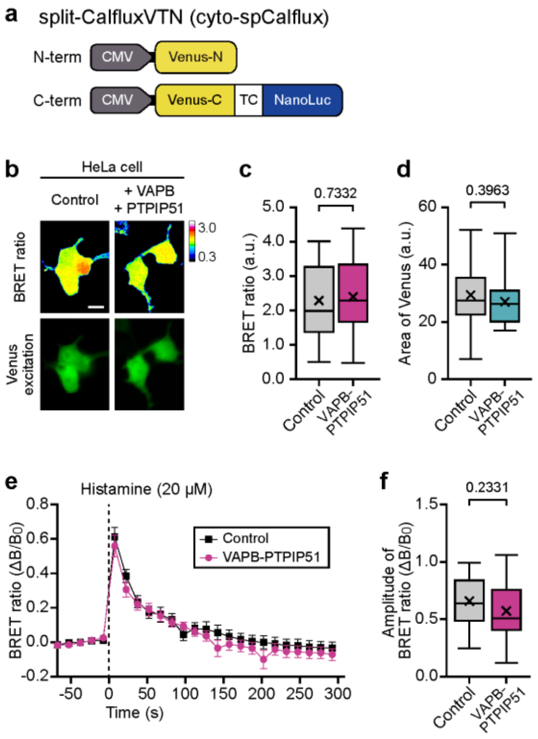

Supplementary Fig. 6. Split-version of CalfluxVTN detected comparable cytosolic Ca^{2+} homeostasis with VAPB-PTPIP51 over-expression.

(a) Schematic diagram of the construct design for cytosolic split-version of CalfluxVTN (cyto-spCalflux). Venus fluorescence domain was separated into two fragments, Venus-N (1-173 aa of Venus, VN173) and Venus-C (156–228 aa of Venus, VC155), equivalent to MAM-Calflux

(b-d) Cyto-spCalflux detected comparable cytosolic Ca^{2+} levels and MAM structure formation upon over-expression of the VAPB-PTPIP51 tethering complex in HeLa cells. Representative images (b) and bar graphs representing BRET ratio (c) and cytosol area (d). (n = 25 for control cells, and n = 22 for VAPB-PTPIP51 expressing cells)

(e-f) Cyto-spCalflux detected comparable histamine-induced cytosolic Ca^{2+} signals with VAPB-PTPIP51 over-expression. Ca^{2+}-responsive BRET signal graph (e) and statistically analyzed peak amplitudes (f). (n = 26 for control HeLa cells and n = 18 for VAPB-PTPIP51 expressing HeLa cells)

The scale bars represent 10 μm. All results are presented as means ± SEM for (e) and box plots

representing the median and interquartile range with whiskers min/max value and the cross representing the mean value for (c-d) and (f). The P-value was calculated using two-tailed Student's t-test for (c-d) and (f). Source data from (c)-(f) are provided as a Source Data file.

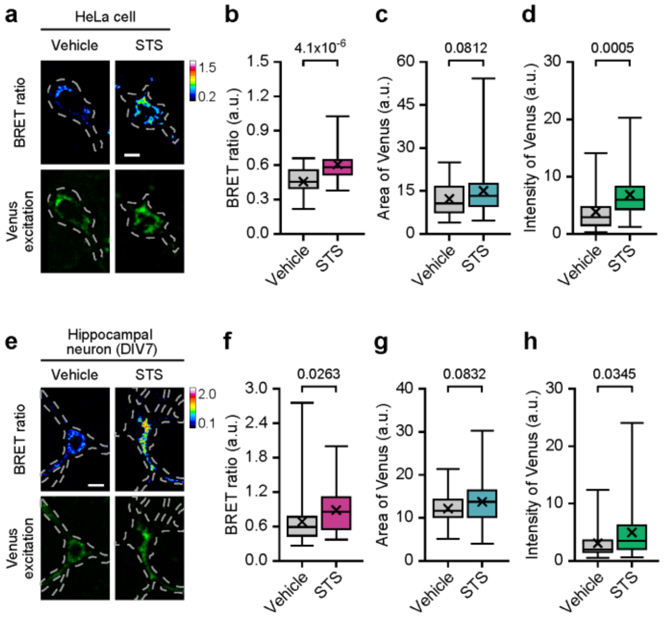

Supplementary Fig. 7. MAM-Calflux measures changes in the steady-state Ca^{2+} level at MAM upon STS treatment.

MAM-Calflux detected the increased MAM Ca^{2+} levels and enhanced MAM structure formation upon 1 μM STS treatment for 2 h in HeLa cells (a-d) and mouse hippocampal neurons (e-h). Representative images (a, e) and bar graphs representing BRET ratio (b, f), MAM area (c, g), and total Venus excitation intensities (d, h). Dashed lines in (a) and (e) represent the cell morphologies. (n = 38 for vehicle-treated HeLa cells, n = 42 for STS-treated HeLa cells, n = 40 for vehicle-treated neurons, and n = 45 for STS-treated neurons)

The scale bars represent 10 μm. All results are presented as box plots representing the median and interquartile range with whiskers min/max value and the cross representing the mean value. All P-values were calculated using two-tailed Student's t-test for (b-d) and (f-h). Source data from (b)-(d) and (f)-(h) are provided as a Source Data file.

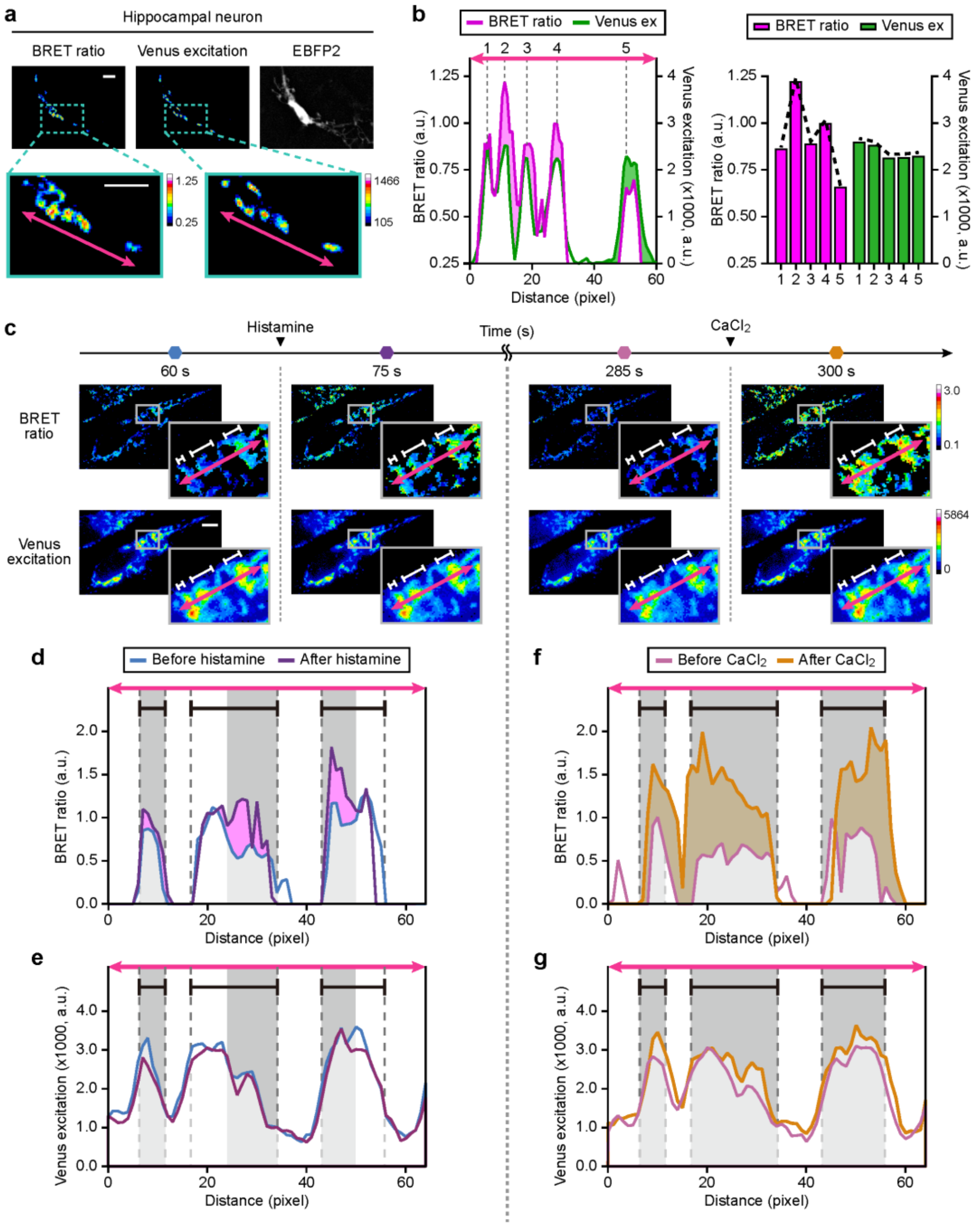

Supplementary Fig. 8. MAM-Calflux can compare the intracellular distribution of MAM calcium levels and MAM structure integrity.

(a-b) The local distribution of MAM Ca^{2+} levels of the primary cultured neurons. Representative images (a) and line profile plots (b) of the BRET ratio (magenta line) and Venus excitation intensities (green line). Magenta line segments in (a) indicate region-of-interest for line profile plots.

(c-g) The intracellular distribution of MAM Ca^{2+} levels (BRET ratio) and MAM structures (Venus excitation) during time-lapse imaging with serial treatments of 10 μM histamine and ionomycin with 1 mM $CaCl_2$. Representative images (c) with the magenta line segments indicating the position for line profile measurement and white line segments indicating MAM regions. Line profile plots of BRET ratio (d, f) and Venus excitation intensities (e, g) before and after the histamine (d-e) and the ionomycin/$CaCl_2$ (f-g). Light gray boxes indicate MAM regions with Ca^{2+} in response to the histamine or $CaCl_2$ treatment. The scale bars represent 20 μm for (a) and 10 μm for (c). Data representative of three experimental repeats. Source data from (b) and (d)-(g) are provided as a Source Data file.

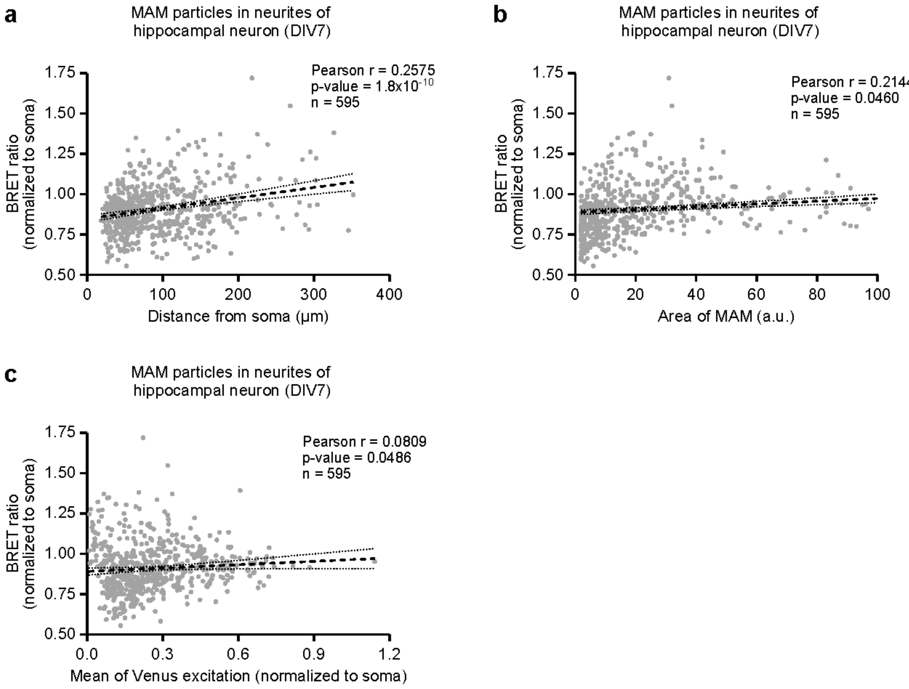

Supplementary Fig. 9. Distribution of normalized BRET signals in MAM puncta in the neurites of DIV7 hippocampal neurons.

(a) Correlation between BRET signals, normalized to mean BRET signals at the soma and distance from the center of the soma. Each dot represents each MAM puncta along the neurites (n = 595 from 27 individual neurons).

(b) Correlation between normalized BRET signals and area of MAM puncta. Each dot represents each MAM puncta along the neurites (n = 595 from 27 individual neurons).

(c) Correlation between normalized BRET signals and mean Venus excitation signals, normalized to the soma. Each dot represents each MAM puncta located at the neurites (n = 595 from 27 individual neurons).

All P-values were calculated using Pearson correlation coefficient analysis with two-tailed P-values. The black dashed lines represent simple linear regression with black dotted lines representing 95% confidence intervals. Source data are provided as a Source Data file.

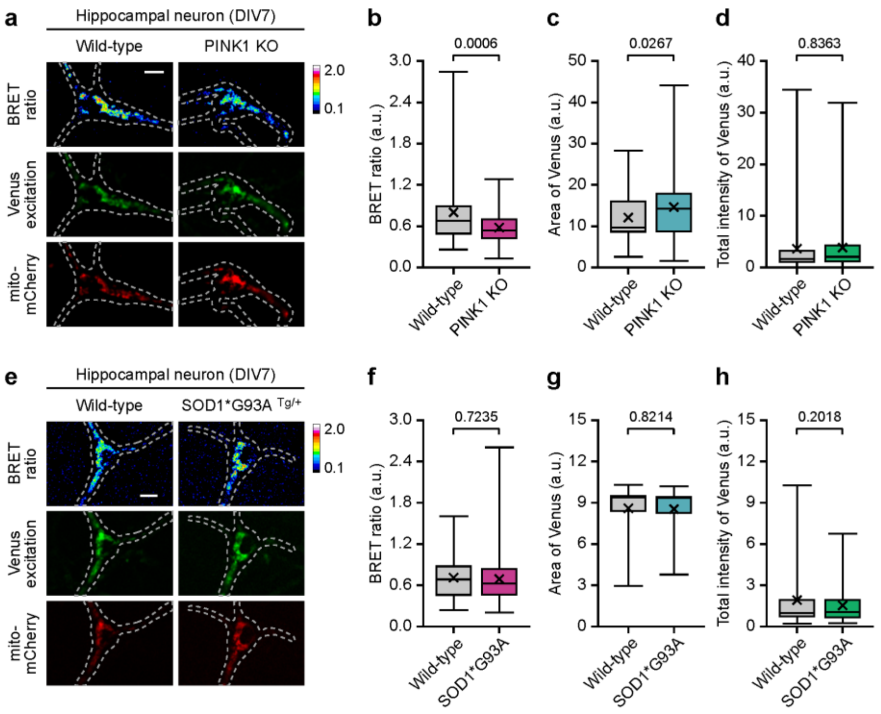

Supplementary Fig. 10. MAM-Calflux can measure differential MAM extent and MAM Ca^{2+} levels in neurodegenerative disease model neurons.

(a-d) MAM-Calflux detected the decreased MAM Ca^{2+} levels in the soma of primary cultured PINK1 knockout (KO) mouse neurons. Representative images (a) and bar graphs representing BRET ratio (b), MAM area (c), and total Venus excitation intensities (d). (n = 57 for wild-type neurons and n = 89 for PINK1 KO neurons)

(e-h) MAM-Calflux detected non-significant change in MAM Ca^{2+} levels in the soma of primary cultured SOD1*G93A tg mouse neurons. Representative images (e) and bar graphs representing BRET ratio (f), MAM area (g), and total Venus excitation intensities (h). (n = 67 for wild-type neurons and n = 80 for SOD1*G93A tg neurons)

Dashed lines in (a) and (e) represent neuronal morphologies. The scale bars represent 10 μm. All results are presented as box plots representing the median and interquartile range with whiskers min/max value and the cross representing the mean value. All P-values were calculated using two-tailed Student's t-test for (b-d) and (f-h). Source data from (b)-(d) and (f)-(h) are provided as a Source Data file.
