# Supplementary material for: Ratiometric measurement of MAM Ca2+ dynamics using a modified CalfluxVTN
Source: Nat Commun. 2023 Jun 16;14:3586. doi: 10.1038/s41467-023-39343-2 (PMC10276021; doi:10.1038/s41467-023-39343-2)
Supplement: Supplementary file 3 — Reporting summary [file 41467_2023_39343_MOESM3_ESM.pdf]

nature portfolio

Corresponding author(s): Sang Ki Park
Youngsik Woo

Last updated by author(s): May 30, 2023

Reporting Summary

Nature Portfolio wishes to improve the reproducibility of the work that we publish. This form provides structure for consistency and transparency in reporting. For further information on Nature Portfolio policies, see our Editorial Policies and the Editorial Policy Checklist.

Statistics

For all statistical analyses, confirm that the following items are present in the figure legend, table legend, main text, or Methods section.

n/a	Confirmed	
☐	☒	The exact sample size (*n*) for each experimental group/condition, given as a discrete number and unit of measurement
☐	☒	A statement on whether measurements were taken from distinct samples or whether the same sample was measured repeatedly
☐	☒	The statistical test(s) used AND whether they are one- or two-sided<br>*Only common tests should be described solely by name; describe more complex techniques in the Methods section.*
☒	☐	A description of all covariates tested
☐	☒	A description of any assumptions or corrections, such as tests of normality and adjustment for multiple comparisons
☐	☒	A full description of the statistical parameters including central tendency (e.g. means) or other basic estimates (e.g. regression coefficient) AND variation (e.g. standard deviation) or associated estimates of uncertainty (e.g. confidence intervals)
☐	☒	For null hypothesis testing, the test statistic (e.g. *F*, *t*, *r*) with confidence intervals, effect sizes, degrees of freedom and *P* value noted<br>*Give P values as exact values whenever suitable.*
☒	☐	For Bayesian analysis, information on the choice of priors and Markov chain Monte Carlo settings
☒	☐	For hierarchical and complex designs, identification of the appropriate level for tests and full reporting of outcomes
☐	☒	Estimates of effect sizes (e.g. Cohen's *d*, Pearson's *r*), indicating how they were calculated

Our web collection on statistics for biologists contains articles on many of the points above.

Software and code

Policy information about availability of computer code

Data collection	MetaMorph 7.7 software (Molecular Devices) was used to obtain BRET images in Fig 2, Fig 3, Fig 4, Fig 5a-d, Fig 5f-i, Fig 6a-f, and Supplementary Fig 5a-f, Supplementary Fig 6, Supplementary Fig 7, Supplementary Fig 8, Supplementary Fig 9, Supplementary Fig 10, and Supplementary Fig 11b-e. FV31S software (Olympus) was used to obtain confocal microscope images in Fig 1d, Fig 5e, Fig 6g-j, Supplementary Fig 1, Supplementary Fig 5g-l, and Supplementary Fig 11a. i-control 1.9 software for Infinite M200 pro (Tecan) was used to obtain wavelength scan data in Fig 1g-h and Supplementary Fig 2.
Data analysis	All BRET images were analyzed by Image J 1.52p(Fiji) software (National Institute of Health) with "Template Matching" and "BRET Analyzer 1.0.7" plug-ins. CellSens 2.3 (Olympus) and Imaris 9.2 softwares were used to analyze confocal microscope images. Statistical analysis was performed with Prism 9.4 (GraphPad).

For manuscripts utilizing custom algorithms or software that are central to the research but not yet described in published literature, software must be made available to editors and reviewers. We strongly encourage code deposition in a community repository (e.g. GitHub). See the Nature Portfolio guidelines for submitting code & software for further information.

March 2021

Data

Policy information about availability of data

All manuscripts must include a data availability statement. This statement should provide the following information, where applicable:

- Accession codes, unique identifiers, or web links for publicly available datasets
- A description of any restrictions on data availability
- For clinical datasets or third party data, please ensure that the statement adheres to our policy

All data are available in the main text or the supplementary materials. Materials will be made available upon request. Source data for all figures are provided with the paper.

Human research participants

Policy information about studies involving human research participants and Sex and Gender in Research.

Reporting on sex and gender	Not Applicable
Population characteristics	Not Applicable
Recruitment	Not Applicable
Ethics oversight	Not Applicable

Note that full information on the approval of the study protocol must also be provided in the manuscript.

Field-specific reporting

Please select the one below that is the best fit for your research. If you are not sure, read the appropriate sections before making your selection.

☒ Life sciences ☐ Behavioural & social sciences ☐ Ecological, evolutionary & environmental sciences

For a reference copy of the document with all sections, see nature.com/documents/nr-reporting-summary-flat.pdf

Life sciences study design

All studies must disclose on these points even when the disclosure is negative.

Sample size	Sample sizes for all statistical evaluations are indicated in Figure legends. Statistical calculations were not used to select sample size. Number of cells per group was chosen based on sample size in calcium measurement experiment with microscopic images which meets or exceed previous studies (Park et al 2017 Cell Reports; Vallese et al 2020 Nature Communications)
Data exclusions	No data were excluded from the analysis.
Replication	To ensure reproducibility, we detailed materials and methods for all the experiments. All experiments were repeated at least three individual sets.
Randomization	All sample images were acquired in random order within the sets and all cells were chosen at the random site.
Blinding	Experimenters were blinded to each group allocation during the image analysis by removing any sample information in file names for all group-to-group comparison experiments (Fig 1g-h, Fig 2, Fig 3, Fig 5, Fig 6, Supplementary Fig 1e, Supplementary Fig 2, Supplementary Fig 5, Supplementary Fig 6, Supplementary Fig 7, and Supplementary Fig 10). All the other experiments were analyzed by automated software with experimenters not blinded to samples.

Reporting for specific materials, systems and methods

We require information from authors about some types of materials, experimental systems and methods used in many studies. Here, indicate whether each material, system or method listed is relevant to your study. If you are not sure if a list item applies to your research, read the appropriate section before selecting a response.

Materials & experimental systems

n/a	Involved in the study	
☐	☒	Antibodies
☐	☒	Eukaryotic cell lines
☒	☐	Palaeontology and archaeology
☐	☒	Animals and other organisms
☒	☐	Clinical data
☒	☐	Dual use research of concern

Methods

n/a	Involved in the study	
☒	☐	ChIP-seq
☒	☐	Flow cytometry
☒	☐	MRI-based neuroimaging

Antibodies

Antibodies used

Anti-TOM20 mouse monoclonal antibodies (Clone# 4F3, Cat# ab56783, Abcam, and Clone# F-10, Cat# sc-17764, Santa Cruz Biotechnology)
anti-Calnexin (Cat# ab22595, Abcam)
anti-Calreticulin (Cat# ab2907, Abcam)
Alexa Fluor 568 or 647 conjugated goat anti-mouse IgG (Cat# A-11004 and Cat# A-21236, Molecular Probes)
Alexa Fluor 647 conjugated goat anti-rabbit IgG (Cat#A-21244, Molecular Probes)

Validation

Anti-TOM20 (Clone# 4F3, Cat# ab56783, Abcam): 151 citations,
Manufacturer's data sheet: validated for mouse/rat/human for WB/IHC/Flow-Cyt/IP/ICC-IF.
(https://www.abcam.com/products/primary-antibodies/tomm20-antibody-4f3-bsa-and-azide-free-ab56783.html)

Anti-TOM20 (Clone# F-10, Cat# sc-17764, Santa Cruz Biotechnology): 570 citations,
Manufacturer's data sheet: validated for mouse/rat/human for WB/IP/IF/IHC/ELISA.
(https://www.scbt.com/p/tom20-antibody-f-10)

anti-Calnexin (Cat# ab22595, Abcam): 291 citations,
Manufacturer's data sheet: validated for mouse/rat/human for WB/ICC-IF/IP.
(https://www.abcam.com/products/primary-antibodies/calnexin-antibody-er-marker-ab22595.html)

anti-Calreticulin (Cat# ab2907, Abcam): 204 citations,
Manufacturer's data sheet: validated for mouse/rat/human for ICC-IF/WB.
(https://www.abcam.com/products/primary-antibodies/calreticulin-antibody-er-marker-ab2907.html)

Eukaryotic cell lines

Policy information about cell lines and Sex and Gender in Research

Cell line source(s)

HeLa ATCC

Authentication

No authentication was performed.

Mycoplasma contamination

All cell lines have been tested negative for mycoplasma contamination.

Commonly misidentified lines
(See ICLAC register)

No commonly misidentified lines were used in this study.

Animals and other research organisms

Policy information about studies involving animals; ARRIVE guidelines recommended for reporting animal research, and Sex and Gender in Research

Laboratory animals

10-16 week-old female mice were used to collect primary cultured neurons of all mouse-lines.
C57BL/6J (wild-type, purchased from Hyochang Science (Daegu, South Korea))
SNCA*A53T (background: C57BL/6J, a gift from Dr. Won-Jong Oh, Korea Brain Research Institute)
PINK1 KO (background: C57BL/6J, a gift from Dr. Xiaoxi Zhuang, Chicago University, and Dr. Sang Myun Park, Ajou University School of Medicine)
5xFAD (background: (C57BL/6 x SJL)F1, a gift from Dr. Kyong-Tai Kim, Pohang University of Science and Technology)
SOD1*G93A (background: (C57BL/6 x SJL)F1, purchased from The Jackson Laboratory, Stock No. 002726)

Wild animals

No wild animal was used in this study.

Reporting on sex

In primary neuron culture experiments, sex of embryo was not considered and all sex was used for experiment.

Field-collected samples

No field collected sample was used in this study.

Ethics oversight

All animal procedures were approved by the Institutional Animal Care and Use Committee (IACUC) of Pohang University of Science

Ethics oversight	and Technology (POSTECH-2022-0085). All experiments were performed in accordance with the approved guidelines.

Note that full information on the approval of the study protocol must also be provided in the manuscript.
